# A Three-Step Process to Isolate Large Quantities of Bioactive Sesquiterpene Lactones from *Cichorium intybus* L. Roots and Semisynthesis of Chicory STLs Standards

**DOI:** 10.3390/ph16050771

**Published:** 2023-05-22

**Authors:** Francesca Ruggieri, Philippe Hance, Bruna Gioia, Alexandre Biela, Pascal Roussel, Jean-Louis Hilbert, Nicolas Willand

**Affiliations:** 1 Univ. Lille, Inserm, Institut Pasteur de Lille, U1177—Drugs and Molecules for Living Systems, F-59000 Lille, France; francesca.ruggieri@univ-lille.fr (F.R.); bruna.gioia@univ-lille.fr (B.G.); alexandre.biela@pasteur-lille.fr (A.B.); nicolas.willand@univ-lille.fr (N.W.); 2 UMRT 1158 BioEcoAgro, Univ. Lille, INRAE, Univ. Liège, Univ. Picardie Jules-Verne, YNCREA, Univ. Artois, Univ. Littoral Côte d’Opale, ICV—Institut Charles Viollette, F-59000 Lille, France; jean-louis.hilbert@univ-lille.fr; 3 Joint Laboratory University of Lille—Florimond-Desprez CHIC41Health, F-59655 Villeuneve d’Ascq, France; 4 Univ. Lille, CNRS, Centrale Lille, Univ. Artois, UMR 8181, Unité de Catalyse et Chimie du Solide (UCCS), F-59000 Lille, France; pascal.roussel@univ-lille.fr

**Keywords:** chicory, sesquiterpene lactones, 11,13-dihydrolactucin, lactucin, plant extraction, semisynthesis of analytical standards

## Abstract

Sesquiterpene lactones (STLs) are a large group of terpenoids most commonly found in plants of the Asteraceae family, e.g., in chicory plants, displaying a wide range of interesting biological activities. However, further studies on the biological potential of chicory-derived STLs and analogues are challenging as only four of these molecules are commercially available (as analytical standards), and to date, there are no published or patented simple extraction–purification processes capable of large-scale STLs isolation. In this work, we describe a novel three-step large-scale extraction and purification method for the simultaneous purification of 11,13-dihydrolactucin (DHLc) and lactucin (Lc) starting from a chicory genotype rich in these STLs and the corresponding glucosyl and oxalyl conjugated forms. After a small-scale screening on 100 mg of freeze-dried chicory root powder, the best results were achieved with a 17 h water maceration at 30 °C. With these conditions, we managed to increase the content of DHLc and Lc, at the same time favoring the hydrolysis of their conjugated forms. On a larger scale, the extraction of 750 g of freeze-dried chicory root powder, followed by a liquid–liquid extraction step and a reversed-phase chromatography, allowed the recovery of 642.3 ± 76.3 mg of DHLc and 175.3 ± 32.9 mg of Lc. The two pure STLs were subsequently used in the context of semisynthesis to generate analogues for biological evaluation as antibacterial agents. In addition, other described chicory STLs that are not commercially available were also synthesized or extracted to serve as analytical standards for the study. In particular, lactucin-oxalate and 11,13-dihydrolactucin-oxalate were synthesized in two steps starting from Lc and DHLc, respectively. On the other hand, 11β,13-dihydrolactucin-glucoside was obtained after a MeOH/H_2_O (70/30) extraction, followed by a liquid–liquid extraction step and a reversed-phase chromatography. Together, this work will help facilitate the evaluation of the biological potential of chicory-derived STLs and their semisynthetic analogues.

## 1. Introduction

Sesquiterpene lactones (STLs) are a large group of naturally occurring terpenoids characterized by a fifteen-carbon (C15) backbone. Commonly found in plants such as chicory and other members of the Asteraceae family, these molecules are accumulated in their leaves and roots as specialized metabolites [1]. Among the identified compounds, lactucin (Lc), lactucopicrin (Lp), 8-deoxylactucin, and the corresponding 11β,13-dihydro (DH) derivatives were the principal ones found [2]. Depending on chicory species, previous studies also reported the presence of 15-glucosyl, as well as 15-oxalates conjugates [3] (Table 1). 

These specialized metabolites can protect the plant, acting as anti-herbivory and antimicrobials or inhibiting the growth of competing plants [1]. In addition, these molecules are also well known to be responsible for the bitter taste of chicory [2]. 

The wide range of biological activities exhibited by STLs is mainly attributed to the α-methylene-γ-lactone group present in their structure [4]. In particular, if present, this moiety can act as a Michael acceptor and react with nucleophiles (sulfhydryl or amino groups) in enzymes, transcription factors, and other proteins, alkylating them irreversibly [5]. 

Of particular interest is that several biological activities have been reported for some chicory-derived STLs. For instance, antimicrobial and antifungal activities were described for lactucopicrin, which showed interesting activity against both *S. aureus* and *P. aeruginosa*, and for 11β,13-dihydrolactucin, which showed promising activity against *P. aeruginosa* and five different strains of *Candida* [6]. Furthermore, an anti-adipogenesis effect both in vivo and in vitro and anticancer activity were reported for lactucin [7,8]. Bischoff et al. found lactucin and lactucopicrin to prevent the growth of the HB3 clone of strain Honduras-1 of *Plasmodium falciparum* [9]. Moreover, lactucin, 11β,13-dihydrolactucin, and lactucopicrin were evaluated for analgesic and sedative properties in mice by Wesołowska et al., and the three compounds showed analgesic effects in both models of nociception used [10]. In addition, Rojas-Silva et al. reported the first findings of leishmanicidal activity of bitter STLs isolated from chicory roots [11]. Recently, lactucin and lactucopicrin were also evaluated as acetylcholinesterase inhibitors [12].

Nevertheless, further studies on the biological potential of chicory-derived STLs and semisynthetic analogues are challenging as only four of these molecules (Lc, DHLc, Lp, and DHLp) are commercially available (as analytical standards), and to date, there are no published or patented simple extraction–purification processes capable of large-scale STLs isolation. 

On this basis, we focused our attention on 11β,13-dihydrolactucin and lactucin large-scale extraction. Indeed, the presence of two hydroxyl groups, an exocyclic double bond, and a ketone in their structure makes these two STLs an interesting starting point for the semisynthesis of other chicory-derived STLs (expensive or not commercially available) and original potentially bioactive analogues.

**Figure 1:**
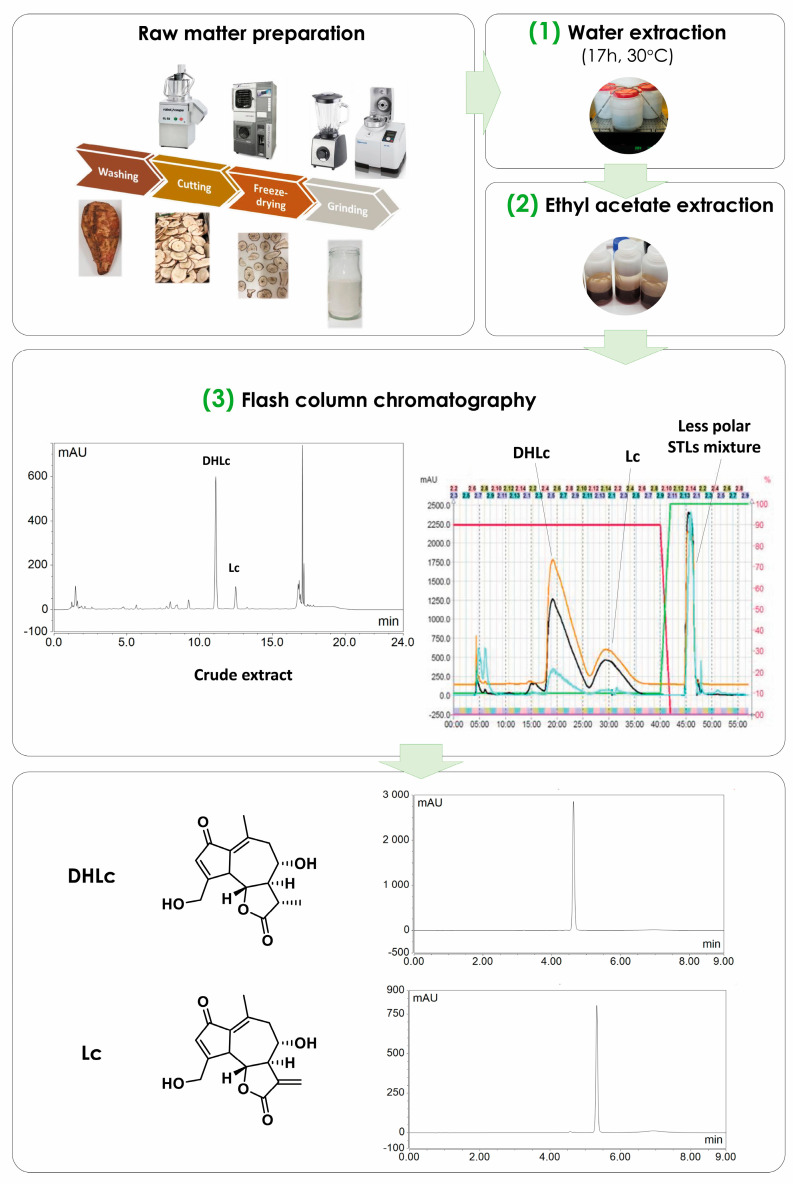
Scheme of the standardized extraction−purification process. After the raw matter preparation, a water maceration of the chicory root powder (**1**) and an ethyl acetate extraction of the supernatant (**2**) are performed. Eventually, a flash column chromatography allows the isolation of pure DHLc and Lc (**3**).

Our presented work describes a novel three-step large-scale extraction and purification method for the simultaneous purification of both 11β,13-dihydrolactucin and lactucin starting from a chicory genotype rich in the corresponding conjugated forms, i.e., 11β,13-dihydrolactucin-glucoside (DHLc-glu), 11β,13-dihydrolactucin-oxalate (DHLC-ox), and lactucin-oxalate (Lc-ox). The process is simple, cheap, and characterized by a limited number of steps and solvents involved (ultra-pure water, ethyl acetate, and acetonitrile) (Figure 1). Eventually, the two extracted STLs were subsequently used in the context of semisynthesis to obtain analogues for biological evaluation as antibacterial agents. In addition, other chicory STLs that are not commercially available were also synthesized to serve as analytical standards, including DHLc-ox and Lc-ox. 

## 2. Results

### 2.1. Identification of the Main STLs 

**Figure 2:**
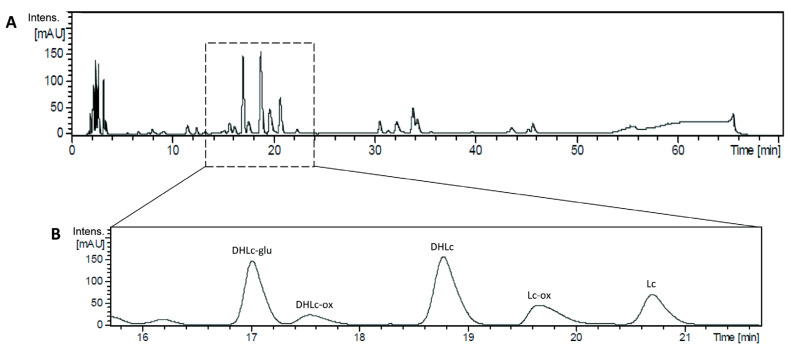
HPLC profile of a water chicory extract (**A**) and zoom on DHLc, Lc, and their conjugated forms (**B**).

At the beginning of our study, before the screening of different parameters to find the best conditions for DHLc and Lc isolation, we wanted to confirm the identity of the two targeted STLs and their conjugated forms by LC-QTOF-HRMS analysis. Back then, a different chicory genotype richer in DHLc and Lc was available for the experiment. A 70 min-gradient allowed a nice separation of the main STLs present in chicory root and showed the rich and complex profile of a water chicory extract (Figure 2). 

The diagnostic fragment ions obtained for the five molecules in positive ionization mode were in accordance with the ones reported in the literature [3,13] (Table 2). DHLc and Lc showed [M+H]^+^ ions at *m*/*z* 279 and 277, respectively, and the [M+Na]^+^ adduct was the most abundant. DHLc-glu showed the [M+H]^+^ ion at *m*/*z* 441, and the [M+Na]^+^ adduct was the most abundant. DHLc-ox and Lc-ox showed [M+H]^+^ ions at *m*/*z* 351 and 349, respectively. For DHLc-ox, the molecular ion [M+H]^+^ was the most abundant. For Lc-ox, the fragment corresponding to the loss of oxaloyl and a molecule of water was the most abundant.

### 2.2. Method Development 

#### Extraction Conditions Determination 

**Figure 3:**
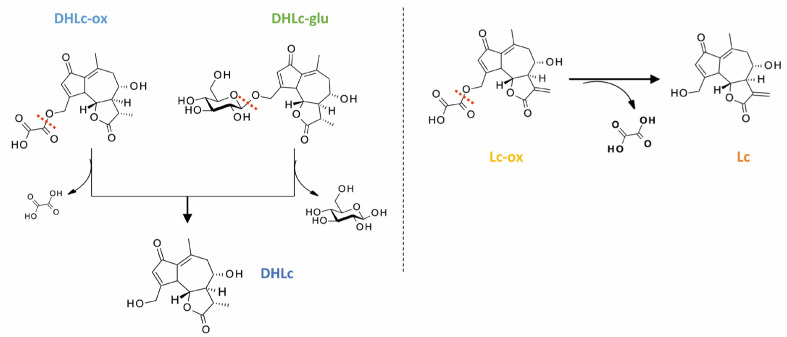
Chemical structures of DHLc, Lc, and their conjugated forms and aimed hydrolysis.

Considering the high quantity of conjugated STLs, i.e., DHLc-glucoside, DHLc-oxalate, and Lc-oxalate, present in our chicory genotype, the aim of our study was to find suitable extraction conditions leading to high concentrations of DHLc and Lc, the two targeted molecules, at the same time favoring the hydrolysis of their conjugated forms (Figure 3). Indeed, oxalyl and glucosyl functionalities can undergo chemical hydrolysis following the addition of a water molecule under different pH conditions. Moreover, we assumed that enzymatic hydrolysis could also take place during the extraction following the action of specific endogenous chicory root enzymes. Interestingly, in support of our theory, few studies in the literature suggested the instability of these conjugated forms after treatment with enzymes or acidified solvents, making their identification in chicory roots extracts difficult [3,13]. 

With this goal in mind, we planned to study the impact of different extraction solvents, times, and temperatures on the level of the five STLs. At the beginning of our study, only commercially available DHLc and Lc were accessible as analytical standards. Therefore, to choose between different extraction conditions, the peak areas of the five STLs measured by UPLC-DAD analysis were compared. Later on, after DHLc-glu extraction-purification (paragraph 2.5) and the synthesis of DHLc-ox and Lc-ox (paragraph 2.6), five calibration curves were performed, and STLs concentrations after different extraction conditions were measured and are reported in Table 3 (means of three samples ± standard deviations). 

**Figure 4:**
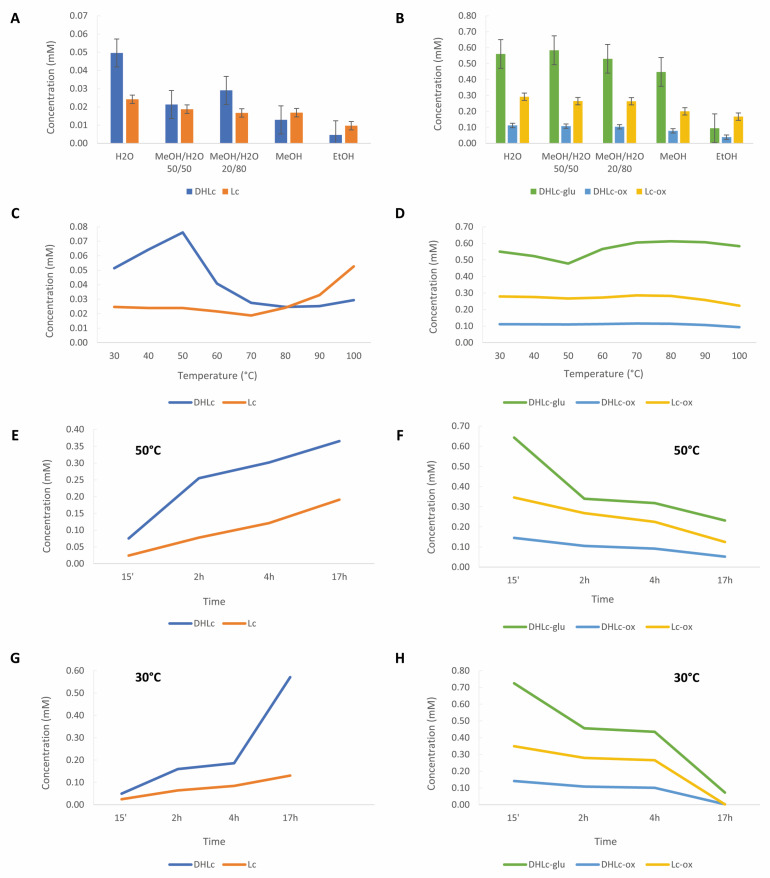
Effect of the extraction solvent on the levels of free (**A**) and conjugated (**B**) STLs. Effect of the extraction temperature on the levels of free (**C**) and conjugated (**D**) STLs. Effect of the extraction time at 50 °C on the levels of free (**E**) and conjugated (**F**) STLs. Effect of the extraction time at 30 °C on the levels of free (**G**) and conjugated (**H**) STLs.

We started our study performing a maceration with the conventional solvents found in the literature. In general, given their hydrophilic features, higher concentrations of the two free STLs were measured using 100% of water (0.050 mM ± 0.003 for DHLc and 0.024 mM ± 0.002 for Lc). With pure methanol and ethanol, the quantity of STLs significantly decreased (0.005 mM ± 0 for DHLc and 0.009 mM ± 0 for Lc) (Figure 4A,B). Methanol/water mixtures efficiently extracted conjugated STLs (Figure 4B) but led to a decrease in DHLc and Lc content (Figure 4A). For instance, the use of water led to an increase in the extraction yield by a factor of 2 for free STLs and no increase for conjugated STLs compared to the use of MeOH/H_2_O 50/50 mixtures. Moreover, the water extraction avoided the presence of polyphenols such as 3-caffeoylquinic acid (**3-CQA**) and 3,5-dicaffeoylquinic acid (**3,5-DiCQA**) whose raw material is rich, normally extracted by alcoholic solvents [14,15,16,17]. These molecules can coelute with the targeted STLs, making the purification step more challenging (Figure S1, supplementary information).

After the selection of water as the best extraction solvent, the effect of the extraction temperature on the levels of the two free molecules and their conjugated forms was studied. Overall, the concentrations of the two free STLs were found to be higher when lower temperatures were chosen. In particular, from 30 °C to 50 °C, the levels of DHLc increased by a factor of 1.5 (from 0.051 mM ± 0.001 to 0.076 mM ± 0.001) (Figure 4C). This phenomenon can be partially explained by the concomitant hydrolysis of DHLc-glu, which decreased by a factor of 1.15 (from 0.550 mM ± 0.006 to 0.478 mM ± 0.010) (Figure 4D). On the contrary, in this temperature range, Lc, Lc-ox, and DHLc-ox concentrations did not increase. Surprisingly, from 60 °C to 80 °C, the DHLc-glu level increased by a factor of 1.08 (from 0.565 mM ± 0.013 to 0.612 mM ± 0.005), and the DHLc level decreased by a factor of 1.64 (from 0.041 mM ± 0 to 0.025 mM ± 0). From 90 °C to 100 °C, a decrease in DHLc-glu and Lc-ox content was found. In contrast, Lc and DHLc concentrations, probably because of the hydrolysis of the corresponding conjugated STLs, started to increase. Despite the interesting levels of Lc observed at 100 °C (increased by a factor of 2.12 compared to 30 °C), these conditions would be too energy consuming for the scale-up of the process. Therefore, 30 °C or 50 °C was considered to be a good compromise for DHLc and Lc extraction and selected for further studies. 

At last, the effect of the extraction time was studied at 50 °C (Figure 4E,F) and 30 °C (Figure 4G,H). The collected data showed that the concentrations of the two free STLs increased with the extraction time, with a maximum at 17 h (increased by a factor of 10 and 5 at 30 °C and 50 °C, respectively, for the sum of DHLc and Lc, compared to 15 min). In contrast, the levels of conjugated molecules dramatically decreased. In particular, compared to 50 °C, 30 °C allowed greater hydrolysis of the conjugated STLs (decrease by a factor of 12 and a factor of 2 at 30 °C and 50 °C, respectively, compared to 15 min). In addition to favoring the hydrolysis of the conjugated forms, the increasing extraction time could also play a role in the extraction efficiency, allowing the solvent to longer penetrate the plant cell walls.

In conclusion, a 17 h maceration by water at 30 °C represented the best condition for DHLc and Lc extraction and for the concomitant hydrolysis of their conjugated forms and was selected for further studies and for the scale-up of the extraction method.

### 2.3. Scaling-Up 

**(1) Water Extraction**. The extraction conditions determined on a small scale (water, 17 h, 30 °C, 1/10 ratio, *w*/*v*) were selected for the scaling-up phase. After the extraction of 750 g of chicory root powder and the centrifugation step, the supernatant was evaporated to directly obtain a crude extract to be purified. 

**(2) Ethyl acetate Extraction**. Because of the large amount of sugars present in chicory roots (e.g., inulin, glucose, fructose, and sucrose), the concentrated sample was caramel-like, very difficult to handle and to use in the further purification step. Therefore, we planned to introduce a liquid–liquid extraction step with a suitable organic solvent. Ethyl acetate, together with chloroform, butanol, and hexanol, was tested. As the collected data show (Table 4), after three ethyl acetate extractions, almost all 11β,13-dihydrolactucin and lactucin passed in the organic phase (recovery rates of 98.8% and 94.8% for DHLc and Lc, respectively). In this way, the crude extract to be purified was poor in sugars and rich in the STLs of interest. 

**(3) Reversed Chromatography**. As for the analytic scale, a Phenyl Butyl (PHC4) reversed phase was used for the purification step. This functionalized silica is very selective for mid-polar compounds and can tolerate up to 100% of water. Its availability also as a flash column allowed an easy transfer of the separation method. The two targeted molecules, together with a mixture of less polar STLs, were obtained. After a freeze-drying step, 642.3 ± 76,3 mg of DHLc and 175.3 ± 32.9 mg of Lc were obtained (means of three extraction–purification cycles), corresponding to a yield of 0.86 ± 0.10 and 0.23 ± 0.04 mg/g dry matter. 

### 2.4. Analytical Validation of STLs 

After flash-chromatography purification, one-dimensional (^1^H-NMR and ^13^C-NMR) and two-dimensional (HMBC and HSQC-DEPT) NMR experiments in DMSO confirmed the structures of the two isolated STLs. Our chemical shifts and assignments are reported in Table 5 and consistent with the ones reported in the literature [18,19]. 

**Figure 5:**
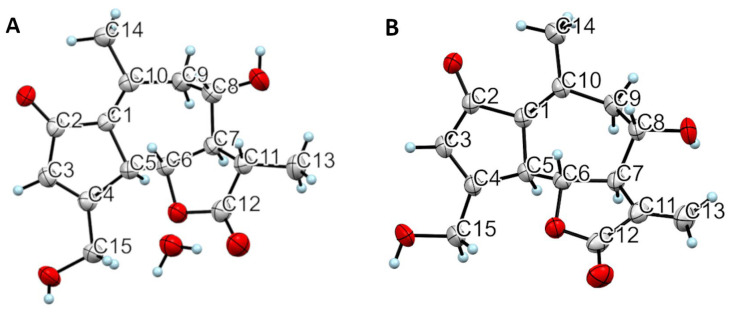
ORTEP drawing of both 11β,13-dihydrolactucin·H_2_O (**A**) and lactucin (**B**).

Moreover, crystallographic studies were performed on single crystals to establish the configuration of the chiral centers and to assure that no racemization occurred during the extraction–purification process (Figure 5). For both DHLc and Lc, the configurations found were consistent with the ones reported in the literature [20,21]. The absolute configurations were assigned to be 5*S*,6*R*,7*R*,8S,11*S* for DHLc and 5*S*,6*R*,7*R*,8*S* for Lc. In the case of DHLc, one molecule of water of crystallization was present.

### 2.5. Isolation of 11β,13-Dihydrolactucin-glucoside

As mentioned above, to determine the content of the main STLs involved in our study after different extraction conditions, we planned to have the five standards available. To the best of our knowledge, 11β,13-dihydrolactucin-glucoside is not commercially available. Therefore, an extraction–purification process was developed. Contrary to the conditions to isolate DHLc and Lc, a short extraction time was chosen to limit the hydrolysis of conjugated STLs, including DHLc-glu, to the corresponding free forms. In addition, to limit the presence of sugars, a MeOH/H_2_O (70/30, *v*/*v*) mixture was chosen. Moreover, to reduce the presence of inulin in the water phase, a precipitation step was introduced after the extraction. Ethanol, well known in the literature, was used [22]. This time, after the liquid–liquid extraction step, the water phase, rich in conjugated STLs, was kept and freeze-dried. After a reversed-phase chromatography, 11β,13-dihydrolactucin-glucoside was obtained as a white powder (13 mg, corresponding to a yield of 3.07 mg/g dry matter).

### 2.6. Synthesis of STLs Standards

**Figure 6:**
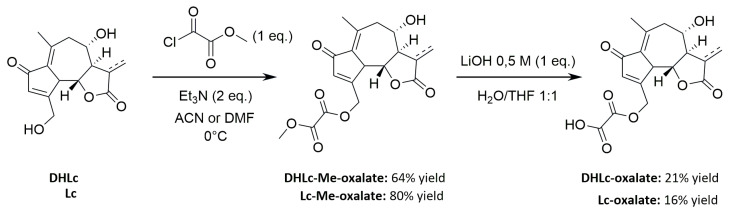
Synthetic strategy towards DHLc-ox and Lc-ox.

Given the low extraction yields and the instability of the conjugated STLs during the extraction process, we thought that a synthetic strategy towards DHLc-ox and Lc-ox would be interesting. Again, to the best of our knowledge, these two conjugated STLs are not commercially available. Starting from extracted DHLc and Lc, 11β,13-dihydrolactucin-oxalate and lactucin-oxalate were synthesized in two steps (Figure 6). 

The first one proceeds with the acylation of the primary alcohol of DHLc/Lc using equimolar quantities of methyl 2-chloro-2-oxo-acetate in the presence of a base (Et_3_N) in acetonitrile (ACN) for DHLc and in *N*,*N*-dimethylformamide (DMF) for Lc (DHLc-Me-oxalate: 64% yield, Lc-Me-oxalate: 80% yield). After optimization, to limit the formation of the di-acylated undesired product, no catalyst was used (i.e., DMAP), and the acyl chloride was diluted and added dropwise at 0 °C to the reaction mixture. In this way, the more reactive primary alcohol can selectively react. For the synthesis of Lc methyl-oxalate, DMF was chosen as the reaction solvent given the poor solubility of Lc in ACN. The second step proceeds with the hydrolysis of the obtained methyl-oxalate derivative with LiOH, leading to the formation of the two lithium salts of DHLc-ox and Lc-ox, which were subsequently purified by reversed-phase preparative HPLC (DHLc-oxalate: 21% yield and Lc-oxalate: 16% yield). Unfortunately, the yields of this second step were low because of the concomitant hydrolysis of the oxalate group and the formation of the starting DHLc and Lc. Therefore, to improve the reaction yield, the optimization of this second step would be required.

## 3. Discussion

In our study, we developed a three-step process for the large-scale extraction and purification of 11β,13-dihydrolactucin and lactucin, starting from a chicory genotype rich in their corresponding conjugated forms. As previously mentioned, these molecules have pharmacological value, together with nutritional (“bittering agents”) and analytical value (HPLC-UV standards). 

Our work addresses the need of producing, at low cost and in a simple manner, these expensive commercially available molecules (EUR 40,000 for one gram of 11β,13-dihydrolactucin [23]). Indeed, to date, there are no publications or patents describing an extraction–purification process that simultaneously targets the two sesquiterpene lactones with a significant productive capacity. The oldest works focus on the discovery and identification of STLs [2,24], while the most recent works look at the biological activity of isolated molecules and/or chicory extracts [6,25]. Overall, these studies do not prioritize the quantity of STLs produced. A few patents focus on lactucin isolation; however, these processes involve a large number of extractions and fractionation steps and a multitude of solvents [26,27,28,29]. 

In our three-step process, starting from freeze-dried chicory roots, an extraction by aqueous maceration is carried out under conditions favorable to the hydrolysis of oxalyl- and glucosyl-conjugated STLs towards the two targeted molecules. Eventually, the two free STLs are obtained simultaneously after a liquid–liquid extraction step followed by reversed-phase flash chromatography (purity greater than 95%). 

Given the existence of more efficient techniques, such as supercritical fluid extractions (SFEs) or continuous extractions, we are aware that the water maceration can be seen as the limiting step of the process, not leading to the best productive capacity. Nevertheless, we think that it represents a really simple and convenient technique to extract important quantities of DHLc and Lc at a laboratory scale, which eventually allows a semisynthetic approach and further structure–activity relationship studies. However, it would be interesting to try to optimize this step by assisting the maceration with ultrasounds. 

The strength of the process is represented by the long extraction time, which leads to the hydrolysis of the conjugated STLs and higher concentrations of targeted DHLc and Lc. Interestingly, in the literature, previous studies suggested the instability of oxalyl and gyclosyl STLs. For instance, Graziani et al. stated that the conversion of glucosides and oxalates to their free forms can be induced by treating a chicory extract with enzymes or acidified methanol [3]. Moreover, Sessa et al. showed that the principal conjugates in *Lactuca* species were not glucosides as frequently reported in earlier studies, but oxalates. The group suggested that in earlier research, these conjugate forms may not have been recovered because of their degradation during the isolation [13]. However, to the best of our knowledge, our study represents the first case where the hydrolysis of conjugated STLs is achieved and observed without the use of enzymes or acidified solvents. 

Thanks to the large quantities isolated, the two STLs were subsequently used in the context of semisynthesis to generate other chicory STLs, which are not commercially available (DHLc-ox and Lc-ox). Moreover, several 11β,13-dihydrolactucin and lactucin analogues were synthesized and will be initially tested for their antibacterial properties. Given this global health challenge [30], our study can open the way to the discovery of new promising hits to fight against antibacterial resistance. In addition, our work can pave the way to the cultivation of industrial chicory as a source of interesting bioactive molecules.

## 4. Materials and Methods

### 4.1. Plant Material

Field-grown chicory roots selected for their high-STLs-content genotype were provided by the company Florimond-Desprez (Cappelle-en-Pévèle, France). The corresponding plants were cultivated in 2020 (Coutiches, France), and the roots were harvested in November from field plot as a pool of 60 roots. They were washed with cold water, cut into slices (robot coupe CL-52, Quiétalis, Tourcoing, France), and freeze-dried (SP VirTis 25 L Genesis, BTF, Diemoz, France). The dried slices were then ground in two steps, with the use of a speed blender in a 1 L stainless steel container (Waring blender, Fisher Scientific, Illkirch, France) followed by an ultra-centrifugal mill (ZM 200 Retsch™, Eragny sur Oise, France) to obtain a fine and homogeneous particle size. The resulting powder was stored at −20 °C.

### 4.2. Reagents and Chemicals

The reagents for the synthesis and the solvents (LC-MS grade) were purchased from commercial suppliers (Fisher Scientific, Sigma Aldrich, France) and used without further purification. Ultra-pure water was obtained from a Milli-Q system (Milli-Q^®^ Advantage A10). The standards, 11β,13-dihydrolactucin and lactucin, were provided by Extrasynthèse (Genay, France). 

### 4.3. Method Development

#### 4.3.1. Extraction of STLs from Freeze-Dried Chicory Roots

Extractions were carried out by maceration with the desired solvent (solid–liquid ratio of 1/10 (g Powder/mL solvent)) on 100 mg of chicory roots using a laboratory shaker (Eppendorf ThermoMixer C, Hamburg, Germany) and an agitation speed of 1000 rpm. The effect of different solvents, temperatures, and times was studied. The solvents used were ultra-pure water, water/methanol mixtures (50/50 and 80/20, *v*/*v*), ethanol, and methanol. For this first investigation, an extraction temperature of 30 °C and an extraction time of 15 min were chosen. Then, the effect of the extraction temperature was studied in the range from 30 °C to 100 °C using ultra-pure water and an extraction time of 15 min. Ultimately, the extraction time was studied in the range from 15 min to 17 h. For each studied parameter, three repetitions were performed. At the end of each extraction, following a centrifugation step (3 min, RCF = 48), the supernatant was separated from the powder deposit and filtered through a 45 µm suitable filter: 0.45 µm PP Whatman UNIFILTER microplate (Cytiva, VWR, Rosny-sous-Bois, France) for aqueous filtration and a syringe-driven filter unit with a polytetrafluoroethylene membrane for organic solvents. Samples were taken to be injected into a UPLC-DAD system to determine the STLs content.

#### 4.3.2. Scaling-Up

##### Selective Fractionation of Free STLs and Removal of Sugars by Liquid–Liquid Extraction

A total of 100 mg of chicory root powder was extracted by water maceration as previously mentioned. Three repetitions were performed. After the centrifugation step (3 min, RCF = 48), 100 µL of the supernatant was kept aside for the following UPLC-DAD analysis, while 500 µL of the supernatant was extracted with ethyl acetate (500 µL). The liquid–liquid extraction step was carried out using a laboratory shaker (Eppendorf ThermoMixer C, Hamburg, Germany) for 10 min at 30 °C and 1000 rpm. The two layers were separated, and the ethyl acetate phase (450 µL) was transferred into another tube and evaporated under vacuum. The residue obtained was then re-dissolved in the same volume of a water/methanol mixture (50/50, *v*/*v*) and kept aside for UPLC-DAD analysis. The remaining water layer was extracted two more times with ethyl acetate following the same procedure. At the end of the experiment, five samples for each repetition were analyzed: the initial filtered water phase, the three ethyl acetate phases, and the final filtered water phase (after the three extractions).

#### 4.3.3. The Standardized Big-Scale Process

Extractions were carried out in a temperature-controlled shaker (Gerhardt THO 500/1, Les Essarts Le Roi, France), and three plastic bins of 5 L were used. A total of 250 g of chicory powder was weighted in each bin, suspended in 2.5 L of ultra-pure water, and agitated for 17 h at 30 °C. After the extraction, the liquid was combined and centrifuged (5 min, RCF = 20,335). A total of 6 L of supernatant was collected and concentrated using an industrial rotavapor (BUCHI Rotavapor^®^ R-220 Pro). The remaining liquid (~1 L) was then extracted three times with ethyl acetate. The liquid–liquid extraction was carried out in 1 L centrifuge bottles. After shaking, a centrifugation step was performed (5 min, RCF = 20,335). The ethyl acetate phases were then combined (~3 L), dried over anhydrous magnesium sulphate, and concentrated under reduced pressure. Finally, a crude extract of 3–4 g was obtained. Two equivalents of celite were then added to prepare the solid deposit for the following reversed-phase chromatography. The obtained powder was loaded into an 80 g PHC4 15 µm F0080 flash column and purified using an Interchim^®^ PuriFlash 4250 system (Interchim, Montluçon France) with 0.1% (*v*/*v*) formic acid in acetonitrile (B) and 0.1% (*v*/*v*) formic acid in water (A) as the mobile phase. The gradient elution was as follows: isocratic at 10% B for 40 min; linear from 10% to 100% B in 2 min; isocratic at 100% B for 3 min; linear from 100% to 50% B in 2 min; and isocratic at 50% B for 3 min. After a freeze-drying step, pure 11β,13-dihydrolactucin and lactucin were obtained as white powders. A total of 642.3 ± 76,3 mg of DHLc and 175.3 ± 32.9 mg of Lc were recovered (means of three extraction–purification cycles), corresponding to a yield of 0.86 ± 0.10 and 0.23 ± 0.04 mg/g dry matter, respectively. DHLc: HRMS (TOF, ES+) *m*/*z* [M+H]^+^: calculated for C_15_H_18_O_5_ 279.1232, found 279.1226. Lc: HRMS (TOF, ES+) *m*/*z* [M+H]^+^: calculated for C_15_H_16_O_5_ 277.1076, found 277.1068.

### 4.4. 11β,13-Dihydrolactucin-glucoside Isolation

A total of 40 g of dried chicory root powder was weighed in a 1 L glass bottle and extracted with a mixture of methanol/water (70/30, *v*/*v*, solid–liquid ratio of 1/10 (g Powder/mL solvent)) in a temperature-controlled shaker (Eppendorf ThermoMixer C, Hamburg, Germany) at 30 °C for 1 h. After the extraction, the liquid was centrifuged (5 min, RCF = 20,335). Ethanol (40%) was added to the supernatant and the mixture was left to stand overnight at a temperature of 4 °C. A liquid–liquid extraction step with ethyl acetate was then carried out as previously described. The water phase was recovered and freeze-dried, and a solid deposit was prepared as already indicated. 11β,13-dihydrolactucin-glucoside was obtained after a reversed-phase chromatography using a 25 g PHC4 15 µm F0025 flash column and an Interchim^®^ PuriFlash 4250 system with 0.1% (*v*/*v*) formic acid in acetonitrile (B) and 0.1% (*v*/*v*) formic acid in water (A) as the mobile phase. The gradient elution was as follows: isocratic at 1% B for 7 min; linear from 1 to 10% B in 11 min; isocratic at 10% B for 17 min; linear from 10 to 37% B in 15 min; linear from 37 to 100% B in 5 min; isocratic at 100% B for 3 min; linear from 100 to 50% B in 2 min; and isocratic at 50% B for 3 min. After a freeze-drying step, pure 11β,13-dihydrolactucin-glucoside was obtained as a white powder (13 mg, corresponding to a yield of 3.07 mg/g dry matter). ^1^H NMR (300 MHz, DMSO) δ 6.57 (s, 1H), 5.22 (m, 2H), 4.97–4.91 (m, 2H), 4.67–4.57 (m, 2H), 4.53–4.48 (m, 1H), 4.23 (d, *J* = 7.7 Hz, 1H), 3.76–3.72 (m, 2H), 3.69–3.64 (m, 1H), 3.59–3.53 (m, 1H), 3.48–3.41 (m, 1H), 3.19–2.99 (m, 4H), 2.73–2.59 (m, 2H), 2.34 (s, 3H), 2.25 (dd, *J* = 13.0, 1.3 Hz, 1H), 2.16–2.09 (m, 1H), and 1.25 (d, *J* = 6.9 Hz, 3H). ^13^C NMR (75 MHz, DMSO) δ 195.01, 178.28, 170.56, 147.84, 133.50, 132.20, 103.28, 80.85, 77.29, 76.85, 74.03, 70.33, 68.39, 68.32, 61.35, 60.43, 48.80, 48.54, 40.99, 21.55, and 15.72. HRMS (TOF, ES+) *m*/*z* [M+H]^+^**:** calculated for C_21_H_28_O_10_ 441.1761, found 441.1758.

### 4.5. STLs Analysis

#### 4.5.1. Quantification of STLs by UPLC-DAD

STLs content was determined using an Ultimate 3000RS system equipped with an LPG-3400RS pump, a WPS-3000TRS autosampler, and DAD-3000RS (Thermo Fisher Scientific, Waltham, MA, USA). Chromatographic separation was achieved on an Uptisphere Strategy PHC4 column (3 µm, 150 × 3 mm: Interchim, Montluçon, France) at 45 °C with 0.1% (*v*/*v*) formic acid in acetonitrile (B) and 0.1% (*v*/*v*) formic acid in water (A) as mobile phases at a flow rate of 0.700 mL/min. The gradient elution was as follows: linear from 6 to 17.5% B in 14 min; linear from 17.5 to 85% B in 1 min; isocratic at 85% B for 2 min; linear from 85 to 6% B in 1 min; and isocratic at 6% B for 6 min. The injection volume was 5.0 μL. The quantification was performed by external calibration using standards (DHLc-glu, DHLc-ox, DHLc, Lc-ox, and Lc). The respective calibration curves were constructed by linear regression plotting signal area versus compound concentration.

#### 4.5.2. LC-QTOF-HRMS Analysis of STLs

STLs were identified using an Ultimate 3000RS system equipped with DAD-3000RS (Thermo Fisher Scientific, Waltham, MA, USA), interfaced with a high-resolution quadrupole time-of-flight mass spectrometer and equipped with an ESI source (Impact II, Bruker Daltonik GmbH, Bremen, Germany). A total of 100 mg of freeze-dried chicory root powder was extracted with 1 mL of ultra-pure water for 1 h at 30 °C at a speed of 1000 rpm (Eppendorf ThermoMixer C, Hamburg, Germany). Following a centrifugation step (3 min, RCF = 48), the supernatant was filtered through a 0.45 µm PP Whatman UNIFILTER microplate (Cytiva, VWR, Rosny-sous-Bois, France) and transferred to a vial for LC-MS analysis. Chromatographic separation was achieved on an Uptisphere Strategy PHC4 column (2.2 µm, 150 × 3 mm; Interchim, Montluçon, France) at 45 °C with 0.1% (*v*/*v*) formic acid in acetonitrile (B) and 0.1% (*v*/*v*) formic acid in water (A) as the mobile phase at a flow rate of 0.5 mL/min. The gradient elution was as follows: isocratic at 2% B for 1 min; linear from 2 to 50% B in 32 min; linear from 50 to 100% B in 5 min; isocratic at 100% B for 7 min; linear from 100 to 2% B in 5 min; and isocratic at 2% B for 7 min. The injection volume was 2.0 μL. The mass spectrometer was operated in full spectral acquisition mode, in the positive mode at 4,5kV with the following parameters: nebulizer gas (N_2_), 2.4 Bar; dry gas (N_2_), 10 mL/min; dry heater, 250 °C; collision cell energy, 11.0 eV; and end plate offset, 500 V. The acquisition was performed in full scan mode in the 90–1200 *m*/*z* range with an acquisition rate of 1 Hz and with mass accuracy < 5 ppm. Software Compass OtofControl 4.1 was used for the operation of the mass spectrometer and for data acquisition. Data were processed by the software Compass DataAnalysis 4.4 (Bruker Daltonik GmbH, Bremen, Germany).

#### 4.5.3. NMR Analysis

NMR spectra were recorded on a Bruker^®^ Avance-300 spectrometer. The results were calibrated to signals from the solvent as an internal reference (e.g., 2.50 (residual DMSO d6) and 39.52 (DMSO d6) ppm for ^1^H and ^13^C NMR spectra, respectively). Chemical shifts (δ) are in parts per million (ppm) downfield from tetramethylsilane (TMS). The assignments were made using one-dimensional (1D) ^1^H and ^13^C spectra and two-dimensional (2D) HSQC-DEPT and HMBC spectra. NMR coupling constants (*J*) are reported in Hertz (Hz), and splitting patterns are indicated as follows: s (singlet), brs (broad singlet), d (doublet), dd (doublet of doublet), ddd (double of doublet of doublet), and m (multiplet).

#### 4.5.4. X-ray Structural Determination

Single-crystal X-ray crystallographic analyses were conducted on 11β,13-dihydrolactucin and lactucin. Diffraction data were obtained using a combination of phi and omega -scans on a Bruker Apex DUO diffractometer equipped with a Photon 3 hybrid pixel area detector, mounted on a four-circle D8 goniometer. Cu Kα radiation (λ = 1.54178 Å) was obtained using an ImuS Incoatec Cu microfocus sealed tube. The data solution was found using SHELXT [31] and refined using SHELXL [32], as implemented in the Olex 2 crystallographic suite for small molecules [33].

### 4.6. Chemistry

Progress of the chemical reactions was monitored by thin-layer chromatography (TLC) and/or by Ultra Performance Liquid Chromatography–Mass Spectrometry (UPLC-MS). TLC was performed using Merck^®^ commercial aluminum sheets coated with silica gel 60 F254. Visualization was achieved by fluorescence quenching under UV light at 254 nm or 215 nm or stained by potassium permanganate and bromocresol green. Purifications were performed by reversed-phase flash chromatography on prepacked columns (Macherey-Nagel^®^ Chromabond) under pressure using a Combiflash^®^ C18 Rf200 instrument and by preparative HPLC Buchi Pure C-380 on an OmniSphere C18 Dynamax (10 µm, 250 mm × 41.4 mm) column. Products were detected by UV absorption at 215 nm and 254 nm. UPLC-MS analysis was performed on an LC-MS Waters ACQUITY UPLC I-Class system equipped with a UPLC I BIN SOL MGR solvent manager, a UPLC I SMP MGR-FTN sample manager, an ACQUITY UPLC I-Class eK PDA Detector photodiode array detector (210–400 nm), and an ACQUITY QDa (Performance) as the mass detector (full scan ESI+/− in the range of 30–1250). An Acquity BEH C18 column (1.7 μm, 50 mm × 2.1 mm) was used for UPLC analysis. The injection volume was 0.5 μL. For a 5 min analysis, the elution was carried out at pH 3.8 from 100% H_2_O/0.1% ammonium formate to 2% H_2_O/98% ACN/0.1% ammonium formate over 3.5 min. A flow rate of 600 µL/min was used. HRMS analysis was performed on an LCT Premier XE Micromass, using a Waters C18 X-Bridge (3.5 μm, 50 mm × 4.6 mm). A gradient starting from 98% H_2_O 5 mM ammonium formate pH = 3.8 and reaching 100% ACN 5 mM ammonium formate pH = 3.8 within 3 min at a flow rate of 1 mL/min was used. 

#### 4.6.1. Chemical Synthesis

##### General Procedure for the Synthesis of DHLc-Me-oxalate and Lc-Me-oxalate

In a 25 mL round bottom flask, 1 eq. of DHLc (250 mg, 0.9 mmol) or Lc (50 mg, 0.18 mmol) was dissolved in either 5 mL of anhydrous ACN (for DHLc) or in 1 mL of DMF (for Lc) (0.18 M). Then, 2 eq. of Et_3_N (250 µL, 1.80 mmol for DHLc and 50.4 µL, 0.36 mmol for Lc) were added. After cooling to 0 °C, a solution of methyl 2-chloro-2-oxo-acetate (82.6 µL, 0.9 mmol, 1 eq. for DHLc and 16.7 µL, 0.18 mmol, 1 eq. for Lc) in anhydrous ACN or DMF (5 mL for DHLc and 1 mL for Lc, 0.18 M) was added dropwise over 5 min under an argon atmosphere. The mixture was stirred for 2 h at 0 °C under argon. Then, Et_3_N (62.6 µL, 0.45 mmol, 0.5 eq. for DHLc and 18 µL, 0.13 mmol, 0.7 eq. for Lc) was added, followed by a solution of methyl 2-chloro-2-oxo-acetate (20.7 µL, 0.23 mmol, 0.25 eq. for DHLc and 5.8 µL, 63 µmol, 0.35 eq. for Lc) in anhydrous ACN or DMF (1.25 mL for DHLc and 350 µL for Lc, 0.18 M). After completion, the reaction mixture was evaporated under reduced pressure. The following purification by reversed-phase chromatography afforded the desired compounds.

**DHLc-Me-oxalate** was obtained as a white powder after purification by reversed-phase chromatography on a 15 g cartridge, with a gradient from 90% of H_2_O to 30/70 of ACN/H_2_O in 20 min (210 mg, 64% yield). ^1^H NMR (300 MHz, DMSO) δ 6.35 (d, *J* = 1.1 Hz, 1H, H-3), 5.42 (dd, *J* = 17.4, 1.6 Hz, 1H, H-15), 5.25 (d, *J* = 5.3 Hz, 1H, OH-8), 5.05 (d, *J* = 16.7 Hz, 1H, H-15’), 3.89–3.73 (m, 5H, H-5, H-6, H-18), 3.63–3.51 (m, 1H, H-8), 2.76–2.60 (m, 2H, H-11), 2.34 (s, 3H, H-14), 2.28 (dd, *J* = 13.6, 1.8 Hz, 1H, H-9), 2.20–2.08 (m, 1H, H-7), 1.26 (d, *J* = 7.0 Hz, 3H, H-13). ^13^C NMR (75 MHz, DMSO) δ 193.81 (Cq, C-2), 177.54 (Cq, C-12), 165.73 (Cq, C-4), 157.07 (Cq, C-17), 156.26 (Cq, C-16), 148.52 (Cq, C-10), 132.69 (CH, C-3), 131.17 (Cq, C-1), 80.07 (CH, C-6), 67.72 (CH, C-8), 64.83 (CH_2_, C-15), 59.84 (CH, C-7), 53.38 (CH_3_-C18), 48.34 (CH_2_, C-9), 48.11 (CH, C-5), 40.42 (CH, C-11), 21.18 (CH_3_, C-14), and 15.17 (CH_3_, C-13). 

**Lc-Me-oxalate** was obtained as a white powder after purification by reversed-phase flash chromatography on a 4 g cartridge, with a gradient from 90% of H_2_O to 30/70 of ACN/H_2_O in 20 min (53 mg, 80%). ^1^H NMR (300 MHz, DMSO) δ 6.37 (d, *J* = 1.3 Hz, 1H, H-3), 6.15 (dd, *J* = 3.0, 1.4 Hz, 1H, H-13), 6.04 (dd, *J* = 3.2, 1.4 Hz, 1H, H-13’), 5.51 (d, *J* = 5.4 Hz, 1H, OH-8), 5.43 (dd, *J* = 17.3, 1.6 Hz, 1H, H-15), 5.09 (d, *J* = 16.8 Hz, 1H, H-15’), 3.98 (d, *J* = 10.0 Hz, 1H, H-5), 3.87–3.70 (m, 5H, H-6, H-8, H-18), 3.16–3.06 (m, 1H, H-7), 2.78 (dd, *J* = 13.5, 10.7 Hz, 1H, H-9), 2.34 (s, 3H, H-14), 2.35–2.28 (m, 1H, H-9). ^13^C NMR (75 MHz, DMSO) δ 194.26 (Cq, C-2), 169.03 (Cq, C-12), 165.86 (Cq, C-4), 157.59 (Cq, C-17), 156.78 (Cq, C-16), 148.62 (Cq, C-10), 138.24 (Cq, C-11), 133.48 (CH, C-3), 131.94 (Cq, C-1), 122.25 (CH_2_, C-13), 80.88 (CH, C-6), 66.83 (CH, C-8), 65.30 (CH_2_, C-15), 56.63 (CH, C-7), 53.90 (CH_3_-C18), 48.92 (CH_2_, C-9), 48.58 (CH, C-5), and 21.74 (CH_3_, C-14).

##### General Procedure for the Synthesis of DHLc-oxalate and Lc-oxalate

A 10 mL round bottom flask was charged with 1 eq. of DHLc-Me-oxalate (150 mg, 0.41 mmol) or Lc-Me-oxalate (80 mg, 0.22 mmol), 1:1 THF:H_2_O (1M), and a magnetic stir bar under ambient atmosphere. After cooling the biphasic mixture to 0 °C, 1 eq. of LiOH (aq.) 0.5 M was added dropwise (823 µL, 0.41 mmol for DHLc-Me-oxalate and 442 µL, 0.22 mmol for Lc-Me-oxalate). The mixture was then vigorously stirred at 0 °C for 10 min. The presence of the acid was confirmed by LC-MS and TLC staining with bromocresol green (blue spot). The crude was then extracted six times with ethyl acetate. The water layer was freeze-dried, and the pure oxalates were obtained after purification by reversed-phase preparative HPLC. 

**DHLc-oxalate** was obtained as a white powder after purification by reversed-phase preparative HPLC, with a gradient from 100% H_2_O (0.1% HCOOH) to 50% ACN (0.1% HCOOH) in 25 min (30 mg, 21%). ^1^H NMR (300 MHz, DMSO) δ 6.27 (d, *J* = 1.2 Hz, 1H, H-3), 5.37 (dd, *J* = 5.4, 1.6 Hz, 1H, H-15), 5.23 (bs, 1H, OH-8), 5.02 (d, *J* = 17.3 Hz, 1H, H-15), 3.84 (d, *J* = 10.0 Hz, 1H, H-5), 3.80–3.75 (m, 1H, H-6), 3.60–3.54 (m, 1H, H-8), 2.72 (dd, *J* = 13.5, 10.9 Hz, 1H, H-9’), 2.68–2.62 (m, 1H, H-11), 2.34 (s, 3H, H-14), 2.27 (dd, *J* = 13.6, 1.8 Hz, 1H, H-9), 2.20–2.12 (m, 1H, H-7), 1.26 (d, *J* = 7.0 Hz, 3H, H-13). ^13^C NMR (75 MHz, DMSO) δ 194.28 (Cq, C-2), 178.06 (Cq, C-12), 166.61 (Cq, C-4), 159.27 (Cq, C-16/17), 149.04 (Cq, C-10), 133.04 (CH, C-3), 131.69 (Cq, C-1), 80.60 (CH, C-6), 68.25 (CH, C-8), 64.75 (CH_2_, C-15), 60.37 (CH, C-7), 48.86 (CH_2_, C-9), 48.66 (CH, C-5), 40.94 (CH, C-11), 21.70 (CH_3_, C-14), and 15.70 (CH_3_, C-13). HRMS (TOF, ES+) *m*/*z* [M+H]^+^**:** calculated for C_17_H_18_O_8_ 351.1080, found 351.1074.

**Lc-oxalate** was obtained as a white powder after purification by reversed-phase preparative HPLC, with a gradient from 100% H_2_O (0.1% HCOOH) to 30% ACN (0.1% HCOOH) in 25 min (12.3 mg, 16%). ^1^H N(300 MHz, DMSO) δ 6.30 (s, 1H, H-3), 6.16 (d, *J* = 1.5 Hz, 1H, H-13), 6.04 (d, *J* = 1.8 Hz, 1H, H-13’), 5.53 (bs, 1H, OH-8), 5.37 (d, *J* = 17.1 Hz, 1H, H-15), 5.03 (d, *J* = 17.4 Hz, 1H, H-15’), 3.98 (d, *J* = 10.0 Hz, 1H, H-5), 3.85–3.80 (m, 1H, H-6), 3.78–3.73 (m, 1H, H-8), 3.16–3.09 (m, 1H, H-7), 2.79 (dd, *J* = 13.5, 10.7 Hz, 1H, H-9’), 2.36 (s, 3H, H-14), 2.33 (dd, *J* = 13.8, 1.8 Hz, 1H, H-9). ^13^C NMR (75 MHz, DMSO) δ 194.25 (Cq, C-2), 169.06 (Cq, C-12), 166.54 (Cq, C-4), 160.32 (Cq, C-17), 159.76 (Cq, C-16), 148.58 (Cq, C-10), 138.26 (Cq, C-11), 133.25 (CH, C-3), 131.96 (Cq, C-1), 122.23 (CH_2_, C-13), 80.90 (CH, C-6), 66.85 (CH, C-8), 64.33 (CH_2_, C-15), 56.66 (CH, C-7), 48.90 (CH_2_, C-9), 48.60 (CH, C-5), and 21.75 (CH_3_, C-14). HRMS (TOF, ES+) *m*/*z* [M+H]^+^**:** calculated for C_17_H_16_O_8_ 349.0923, found 349.0918.

### 4.7. Statistical Assays

All extractions were performed in triplicate. Data are expressed as the means ± standard deviations of each extraction condition. The assumptions of normality, homogeneity of variance, and independence were checked by the Shapiro–Wilk test, Bartlett test, and Durbin–Watson test, respectively. Significant differences in different extraction conditions on total free and conjugated STLs concentrations were determined using ANOVA followed by Tukey’s or based on the marginal means post hoc test with adjustment by Holm’s or the Bonferroni method. The significance threshold was set to 0.05 for all statistical tests. All analyses were performed with R Statistics 4.2.2. (R Core Team (2017). R: A language and environment for statistical computing. R Foundation for Statistical Computing, Vienna, Austria. URL https://www.R-project.org/ (accessed on the 22 March 2023).

## 5. Conclusions

The experiments carried out in this paper have shown that the optimal extraction operating conditions to increase the content of 11β,13-dihydrolactucin and lactucin were obtained within a 17 h water maceration at a temperature of 30 °C. With these conditions, starting from a chicory genotype rich in conjugated STLs, we managed to decrease their concentration, favoring the liberation of the corresponding free STLs. This result was obtained without employing enzymes or acidifying solvents as previously reported in the literature [3,24]. On this basis, a big-scale process was developed and allowed us to obtain 642.3 ± 76.3 mg of DHLc and 175.3 ± 32.9 mg of Lc (means of three extraction–purification cycles). To date, these two STLs can be bought only from two providers (Extrasynthese and Phytolab) and are quite expensive (EUR 40,000 for one gram of 11β,13-dihydrolactucin). Our process is simple, economical, and characterized by a limited number of steps (three main) and solvents involved (ultra-pure water, ethyl acetate, and acetonitrile). The two extracted molecules were used in the context of semisynthesis to generate potential antibacterial analogues. Moreover, 11β,13-dihydrolactucin-oxalate and lactucin-oxalate were synthesized and used as analytical standards. 11β,13-dihydrolactucin-glucoside was extracted from chicory roots and also used as the analytical standard. Additional studies considering the nature of the hydrolysis of conjugated STLs (enzymatic or chemical) and the peculiar behavior of STLs towards different extraction temperatures would be interesting. 

## Figures and Tables

**Table 1:** Main STLs found in chicory roots.

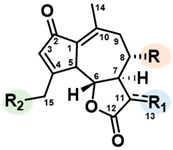			
	R	R_1_	R_2_
**Lactucin**	OH	CH_2_	OH
Lactucin-15-oxalate	OH	CH_2_	OCOCOOH
Lactucin-15-glucoside	OH	CH_2_	OGlucose
**11β,13-dihydrolactucin**	OH	CH_3_	OH
11β,13-dihydrolactucin-15-oxalate	OH	CH_3_	OCOCOOH
11β,13-dihydrolactucin-15-glucoside	OH	CH_3_	OGlucose
**8-deoxylactucin**	H	CH_2_	OH
8-deoxylactucin-15-oxalate	H	CH_2_	OCOCOOH
8-deoxylactucin-15-glucoside	H	CH_2_	OGlucose
11β,13-dihydro-8-deoxylactucin	H	CH_3_	OH
11β,13-dihydro-8-deoxylactucin-oxalate	H	CH_3_	OCOCOOH
11β,13-dihydro-8-deoxylactucin-glucoside	H	CH_3_	OGlucose
**Lactucopicrin**	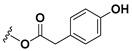	CH_2_	OH
Lactucopicrin-15-oxalate		CH_2_	OCOCOOH
Lactucopicrin-15-glucoside		CH_2_	OGlucose
**11β,13-dihydrolactucopicrin**	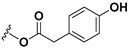	CH_3_	OH
11β,13-dihydrolactucopicrin-15-oxalate		CH_3_	OCOCOOH
11β,13-dihydrolactucopicrin-15-glucoside		CH_3_	OGlucose

**Table 2:** Molecular fragments of STLs determined by positive LC-QTOF-HRMS analysis.

Compound	Molecular Weight	Retention Time (min)	ESI Major Fragment Ions (*m*/*z*)
DHLc-glu	440	16.9	463 (100) [M+Na]^+^; 441 (13) [M+H]^+^; 279 (18) [M-glucosyl+H]^+^; 261 (9) [M-glucosyl-H_2_O+H]^+^; 243 (10) [M-glucosyl-2H_2_O+H]^+^; 215 (8) [M-glucosyl-H_2_O-HCO_2_H+H]^+^
DHLc-ox	350	17.7	373 (57) [M+Na]^+^; 351 (100) [M+H]^+^; 261 (94) [M-oxaloyl-H_2_O+H]^+^; 243 (56) [M-oxaloyl-2H_2_O+H]^+^; 215 (39) [M-oxaloyl-H_2_O-HCO_2_H+H]^+^
DHLc	278	18.8	301 (100) [M+Na]^+^; 279 (59) [M+H]^+^; 261 (7) [M-H_2_O+H]^+^; 243 (8) [M-2H_2_O+H]^+^; 215 (17) [M-HCO_2_H-H_2_O+H]^+^
Lc-ox	348	19.7	371 (73) [M+Na]^+^; 349 (86) [M+H]^+^; 259 (100) [M-oxaloyl-H_2_O+H]^+^; 241 (96) [M-oxaloyl-2H_2_O+H]^+^; 213 (60) [M-oxaloyl-H_2_O-HCO_2_H+H]^+^
Lc	276	20.7	299 (100) [M+Na]^+^; 277 (44) [M+H]^+^; 259 (8) [M-H_2_O+H]^+^; 241 (17) [M-2H_2_O+H]^+^; 213 (18) [M-HCO_2_H-H_2_O+H]^+^

**Table 3:** Content of DHLc-glu, DHLc-ox, DHLc, Lc-ox, and Lc extracted under different conditions. Values are means (±standard deviations) of three samples. For solvent and temperature, different letters in the same column within the same parameters indicate significant differences (*p* < 0.05). For time, statistical differences between 30 °C and 50 °C are noted as ns, not significant, * *p* < 0.05, ** *p* < 0.01, *** *p* < 0.001, and **** *p* < 0.00 and reported in the 30°C rows as result of pair comparison.

STLs Concentration (mM) ± SD								
Parameter		DHLc-glu	DHLc-ox	DHLc	Lc-ox	Lc	TOTAL Conjugated STLs	TOTAL Free STLs
**Solvent** (15’, 30 °C)	H_2_O	0.561 ± 0.012	0.112 ± 0.004	0.050 ± 0.003	0.292 ± 0.008	0.024 ± 0.002	0.965 ± 0.023 *c*	0.0737 ± 0.005 *d*
	MeOH/H_2_O 20/80	0.530 ± 0.099	0.104 ± 0.019	0.029 ± 0.004	0.264 ± 0.048	0.017 ± 0.004	0.898 ± 0.167 *bc*	0.0457 ± 0.008 *c*
	MeOH/H_2_O 50/50	0.584 ± 0.010	0.108 ± 0.003	0.021 ± 0.001	0.265 ± 0.005	0.019 ± 0.002	0.957 ± 0.018 *c*	0.0400 ± 0.003 *bc*
	MeOH	0.448 ± 0.029	0.078 ± 0.005	0.013 ± 0.000	0.201 ± 0.016	0.017 ± 0.003	0.727 ± 0.049 *b*	0.0297 ± 0.003 *b*
	EtOH	0.095 ± 0.002	0.039 ± 0.006	0.005 ± 0.000	0.167 ± 0.005	0.009 ± 0.000	0.301 ± 0.008 *a*	0.0143 ± 0.000 *a*
**Temperature** (15’, H_2_O)	30 °C	0.550 ± 0.006	0.112 ± 0.002	0.051 ± 0.001	0.279 ± 0.006	0.025 ± 0.001	0.940 ± 0.011 *ab*	0.076 ± 0.001 *a*
	40 °C	0.523 ± 0.011	0.111 ± 0.004	0.064 ± 0.001	0.276 ± 0.008	0.024 ± 0.000	0.909 ± 0.023 *bc*	0.088 ± 0.001 *b*
	50 °C	0.478 ± 0.010	0.110 ± 0.001	0.076 ± 0.001	0.267 ± 0.003	0.024 ± 0.000	0.855 ± 0.015 *d*	0.100 ± 0.001 *c*
	60 °C	0.565 ± 0.013	0.112 ± 0.002	0.041 ± 0.000	0.272 ± 0.005	0.022 ± 0.001	0.950 ± 0.014 *ac*	0.062 ± 0.002 *d*
	70 °C	0.605 ± 0.011	0.116 ± 0.004	0.028 ± 0.000	0.286 ± 0.004	0.019 ± 0.000	1.007 ± 0.022 *e*	0.046 ± 0.001 *e*
	80 °C	0.612 ± 0.005	0.114 ± 0.002	0.025 ± 0.000	0.282 ± 0.004	0.024 ± 0.000	1.009 ± 0.006 *e*	0.049 ± 0.001 *e*
	90 °C	0.607 ± 0.013	0.107 ± 0.003	0.025 ± 0.000	0.258 ± 0.002	0.033 ± 0.001	0.971 ± 0.017 *ae*	0.058 ± 0.001 *f*
	100 °C	0.583 ± 0.008	0.093 ± 0.002	0.029 ± 0.001	0.223 ± 0.002	0.053 ± 0.053	0.900 ± 0.008 *bd*	0.082 ± 0.001 *g*
**Time** (H_2_O, 30 °C)	15’	0.552 ± 0.005	0.113 ± 0.026	0.049 ± 0.006	0.291 ± 0.066	0.024 ± 0.003	0.955 ± 0.015 *	0.074 ± 0.026 ns
	2 h	0.456 ± 0.039	0.108 ± 0.008	0.160 ± 0.016	0.280 ± 0.022	0.064 ± 0.007	0.844 ± 0.069 **	0.224 ± 0.023 ***
	4 h	0.435 ± 0.048	0.101 ± 0.010	0.185 ± 0.011	0.265 ± 0.022	0.084 ± 0.008	0.801 ± 0.080 ***	0.270 ± 0.019 ****
	17 h	0.072 ± 0.013	0.003 ± 0.000	0.571 ± 0.066	0.002 ± 0.000	0.131 ± 0.006	0.077 ± 0.014 ****	0.701 ± 0.070 ****
**Time** (H_2_O, 50 °C)	15’	0.485 ± 0.014	0.110 ± 0.002	0.075 ± 0.001	0.269 ± 0.006	0.024 ± 0.001	0.864 ± 0.019	0.100 ± 0.001
	2 h	0.340 ± 0.017	0.105 ± 0.004	0.255 ± 0.008	0.268 ± 0.014	0.078 ± 0.004	0.712 ± 0.035	0.333 ± 0.012
	4 h	0.318 ± 0.016	0.091 ± 0.001	0.302 ± 0.003	0.225 ± 0.003	0.121 ± 0.003	0.634 ± 0.020	0.423 ± 0.003
	17 h	0.231 ± 0.011	0.052 ± 0.003	0.366 ± 0.015	0.125 ± 0.008	0.191 ± 0.011	0.408 ± 0.021	0.556 ± 0.025

**Table 4:** Content of DHLc and Lc after three ethyl acetate extractions. Values are means (± standard deviations) of three samples.

	STLs Concentration (mM) ± SD	
	DHLc	Lc
**Initial H_2_O**	0.571 ± 0.066	0.134 ± 0.005
**EtOAc 1**	0.345 ± 0.010	0.081 ± 0.002
**EtOAc 2**	0.153 ± 0.004	0.033 ± 0.003
**EtOAc 3**	0.066 ± 0.000	0.013 ± 0.011
**Final** **H_2_O**	0.004 ± 0.016	0.005 ± 0.003

**Table 5:** ^1^H and ^13^C NMR data for DHLc and Lc.

	DHLc: (CD_3_)_2_SO		Lc: (CD_3_)_2_SO	
Position	^1^H (mult, *J* Hz)	^13^C	^1^H (mult, *J* Hz)	^13^C
1	-	132.0	-	132.3
2	-	194.9	-	194.8
3	6.27 (s)	132.5	6.28 (d, 1.0)	132.7
4	-	175.8	-	169.2
5	3.76–3.64 (m)	48.4	3.83–3.67 (m)	48.3
6	3.76–3.64 (m)	80.9	3.83–3.67 (m)	81.1
7	2.17–2.05 (m)	60.5	3.12–3.03 (m)	56.8
8	3.61–3.49 (m)	68.4	3.83–3.67 (m)	66.9
9	9: 2.74–2.55 (m)	48.7	9: 2.74–2.55 (m)	48.8
	9’: 2.24 (dd, 13.6, 1.8)		9’: 2.24 (dd, 13.6, 1.8)	
10	-	147.5	-	147.0
11	2.74–2.55 (m)	40.9	-	138.4
12	-	178.3	-	175.4
13	1.25 (d, 7.0)	15.7	13: 6.13 (dd, 3.0, 1.5)	121.9
			13’: 6.01 (dd, 3.2, 1.5)	
14	2.33 (s)	21.5	2.33 (s)	21.5
15	15: 4.64 (ddd, 18.8, 5.8, 1.9)	61.8	15: 4.66 (d, 18.7)	61.8

## Data Availability

Data is contained within the article and Supplementary Material.

## Supplementary Materials

The following supporting information can be downloaded at: https://www.mdpi.com/article/10.3390/ph16050771/s1, NMR spectra of DHLc, Lc, DHLc-Me-oxalate, Lc-Me-oxalate, DHLc-oxalate, Lc-oxalate, and DHLc-glucoside; Figure S1: HPLC profiles (254 and 320 nm) of a water/methanol 50/50 and pure water extracts and different contents in 3-CQA; Figure S2: Base peak chromatogram (BPC, All—MS) of a water chicory extract obtained in the positive mode; diagnostic fragment ions in the positive mode for DHLc-glu, DHLc-ox, DHLc, Lc-ox, and Lc.

## Abbreviations

ACN, acetonitrile; DHLc, 11,13-dihydrolactucin; DHLc-ox, 11,13-dihydrolactucin-oxalate; DHLc-glu, 11,13-dihydrolactucin-glucosyde; DHLp, 11,13-dihydrolactucopicrin; DMF, ***N,N***-dimethylformamide; DMSO, dimethylsulfoxide; DMAP, 4-dimethylaminopyridine; Et_3_N, triethylamine; EtOAc, ethyl acetate; eq, equivalent; LiOH, lithium hydroxide; MeOH, methanol; SFEs, supercritical fluid extractions; STLs, sesquiterpene lactones; THF, tetrahydrofuran.
