# Supplementary material for: A Three-Step Process to Isolate Large Quantities of Bioactive Sesquiterpene Lactones from Cichorium intybus L. Roots and Semisynthesis of Chicory STLs Standards
Source: Pharmaceuticals (Basel). 2023 May 22;16(5):771. doi: 10.3390/ph16050771 (PMC10222082; doi:10.3390/ph16050771)

Supplementary Information

A Three-Step Process to Isolate Large Quantities of Bioactive Sesquiterpene Lactones from *Cichorium intybus* L. Roots and Semisynthesis of Chicory STLs Standards

Francesca Ruggieri [1,†], Philippe Hance [2,3,*,†], Bruna Gioia [1], Alexandre Biela [1], Pascal Roussel [4], Jean-Louis Hilbert [2] and Nicolas Willand [1]

1 Univ. Lille, Inserm, Institut Pasteur de Lille, U1177—Drugs and Molecules for Living Systems, F-59000 Lille, France
2 UMRT 1158 BioEcoAgro, Univ. Lille, INRAE, Univ. Liège, Univ. Picardie Jules-Verne, YNCREA, Univ. Artois, Univ. Littoral Côte d'Opale, ICV—Institut Charles Viollette, F-59000 Lille, France
3 Joint Laboratory University of Lille—Florimond-Desprez CHIC41Health, F-59655 Villeuneve d'Ascq, France
4 Univ. Lille, CNRS, Centrale Lille, Univ. Artois, UMR 8181, Unité de Catalyse et Chimie du Solide (UCCS), F-59000 Lille, France
* Correspondence: philippe.hance@univ-lille.fr
† These authors contributed equally to this work.

^{1}H and ^{13}C NMR Spectra:

11β,13-dihydrolactucin (DHLc), ^{1}H NMR (300 MHz, DMSO)

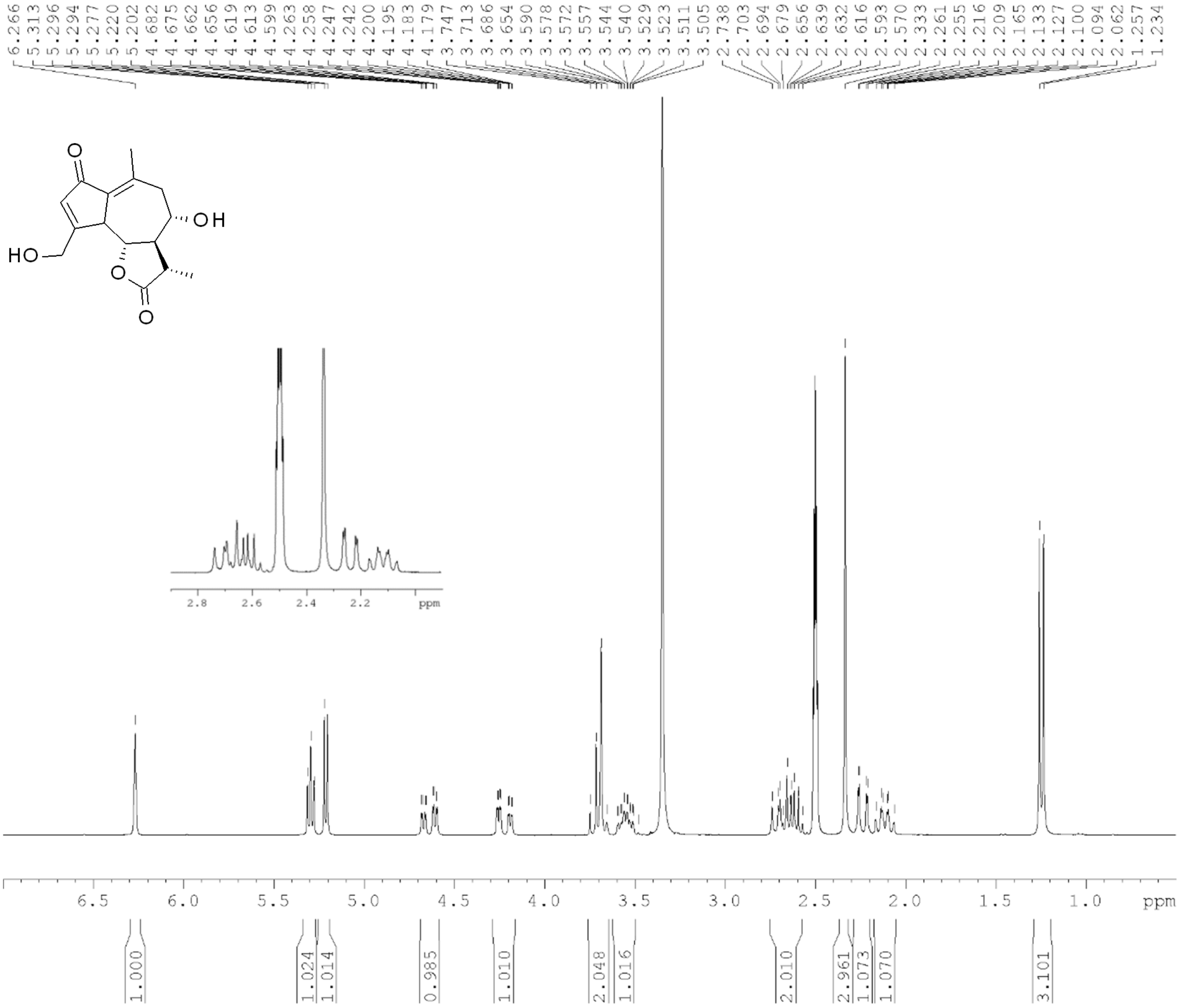

11β,13-dihydrolactucin (DHLc), ^{13}C NMR (75 MHz, DMSO)

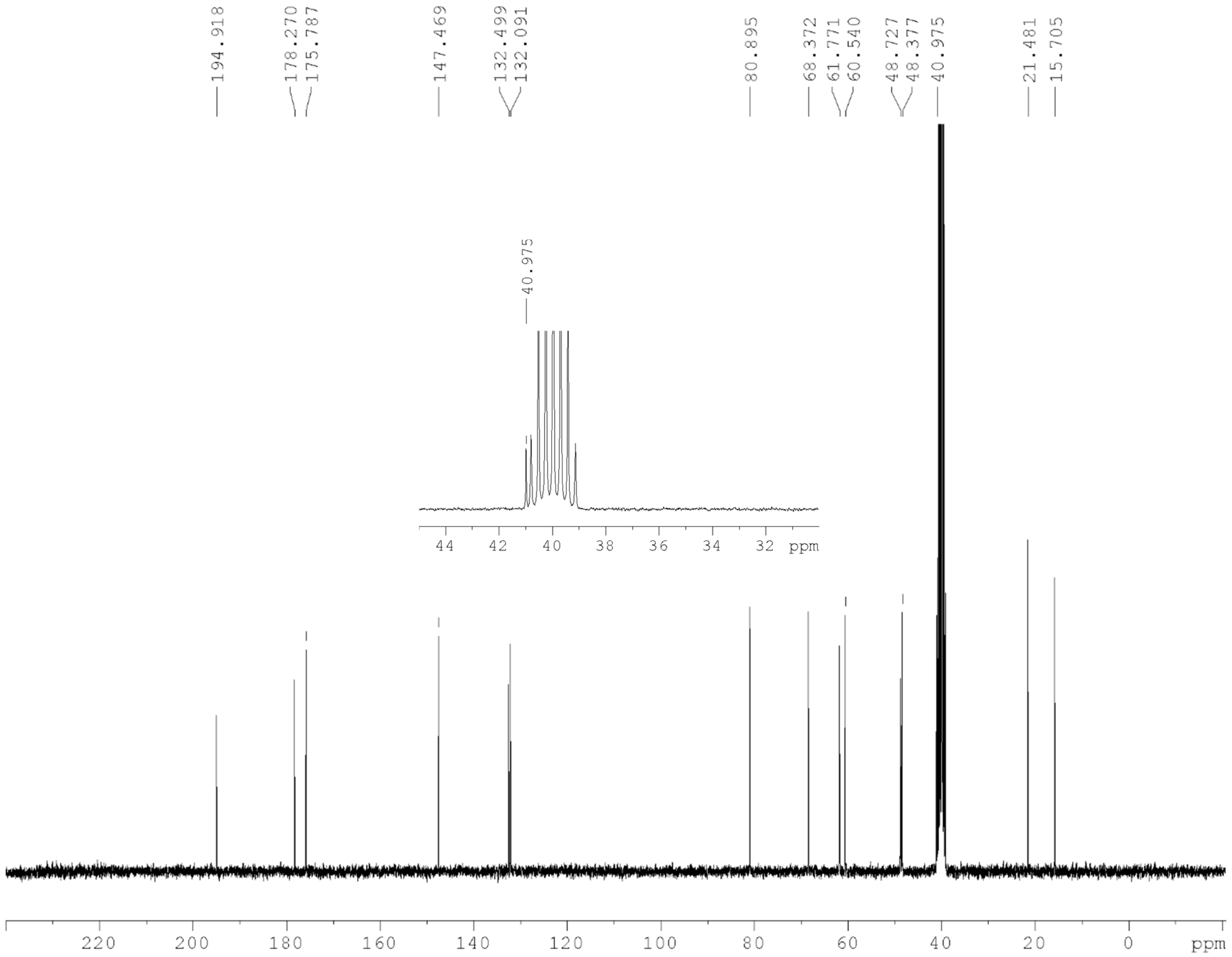

Lactucin (Lc), ^{1}H NMR (300 MHz, DMSO)

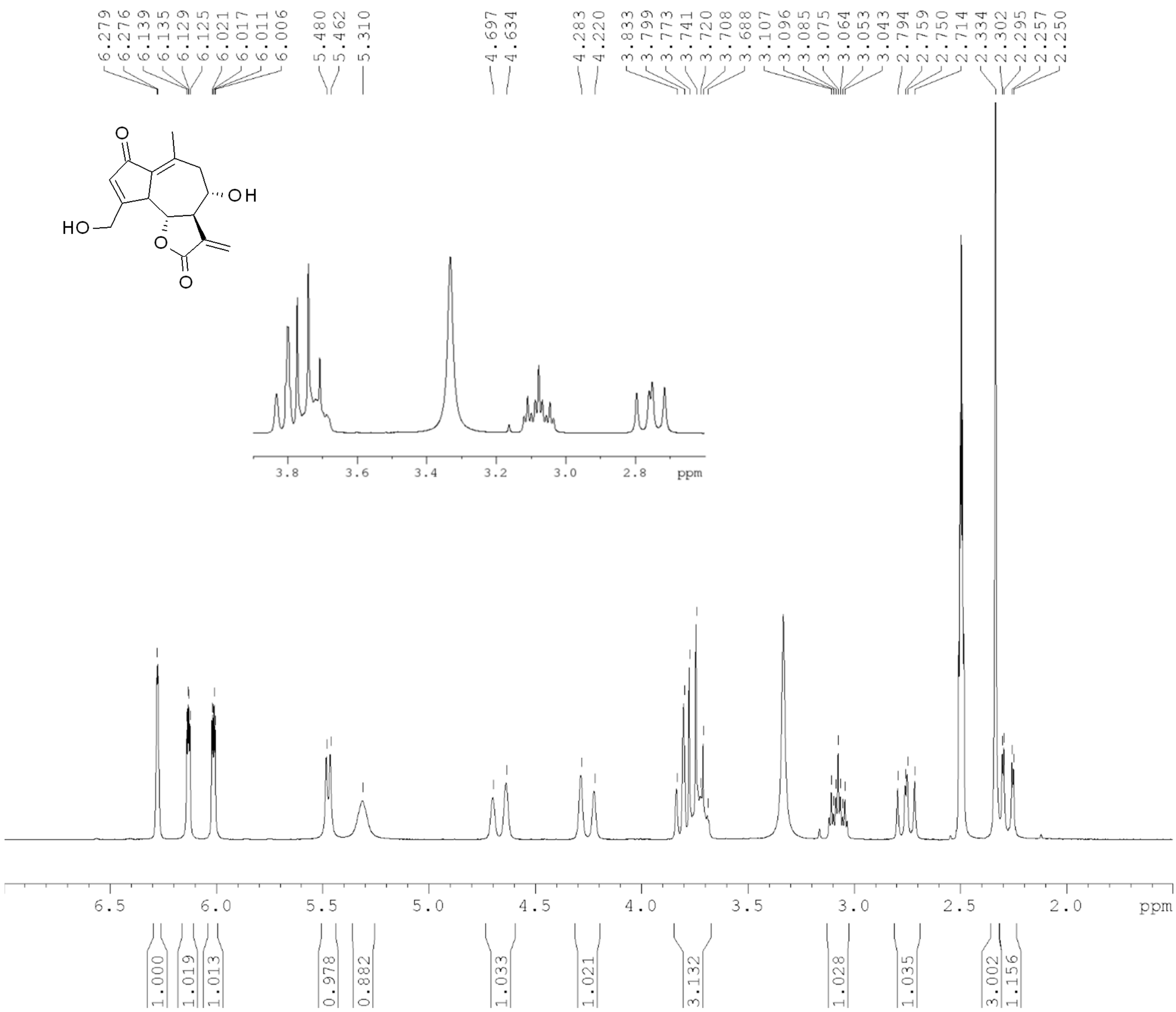

Lactucin (DHLc), ^{13}C NMR (75 MHz, DMSO)

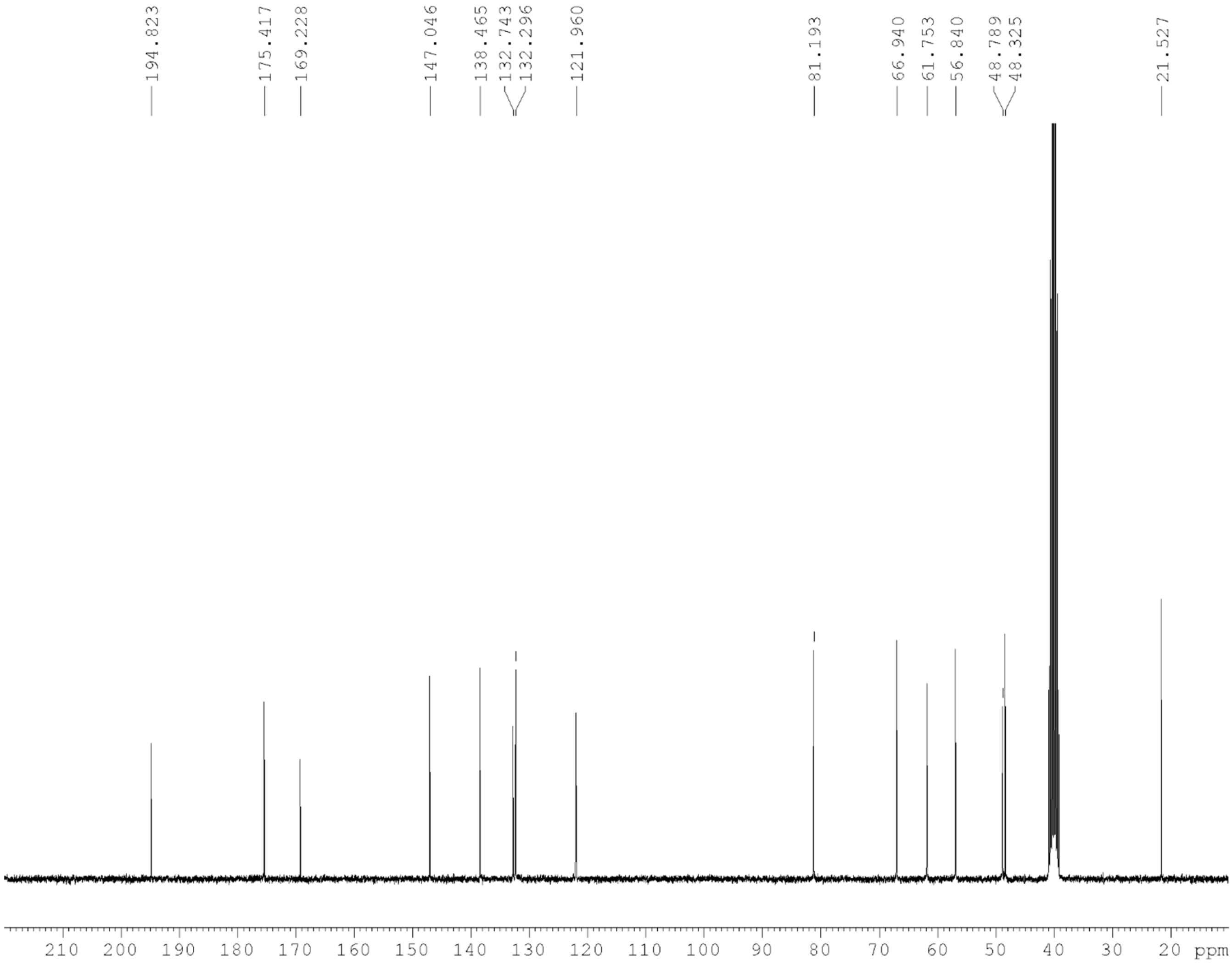

DHLc-Me-oxalate, ^{1}H NMR (300 MHz, DMSO)

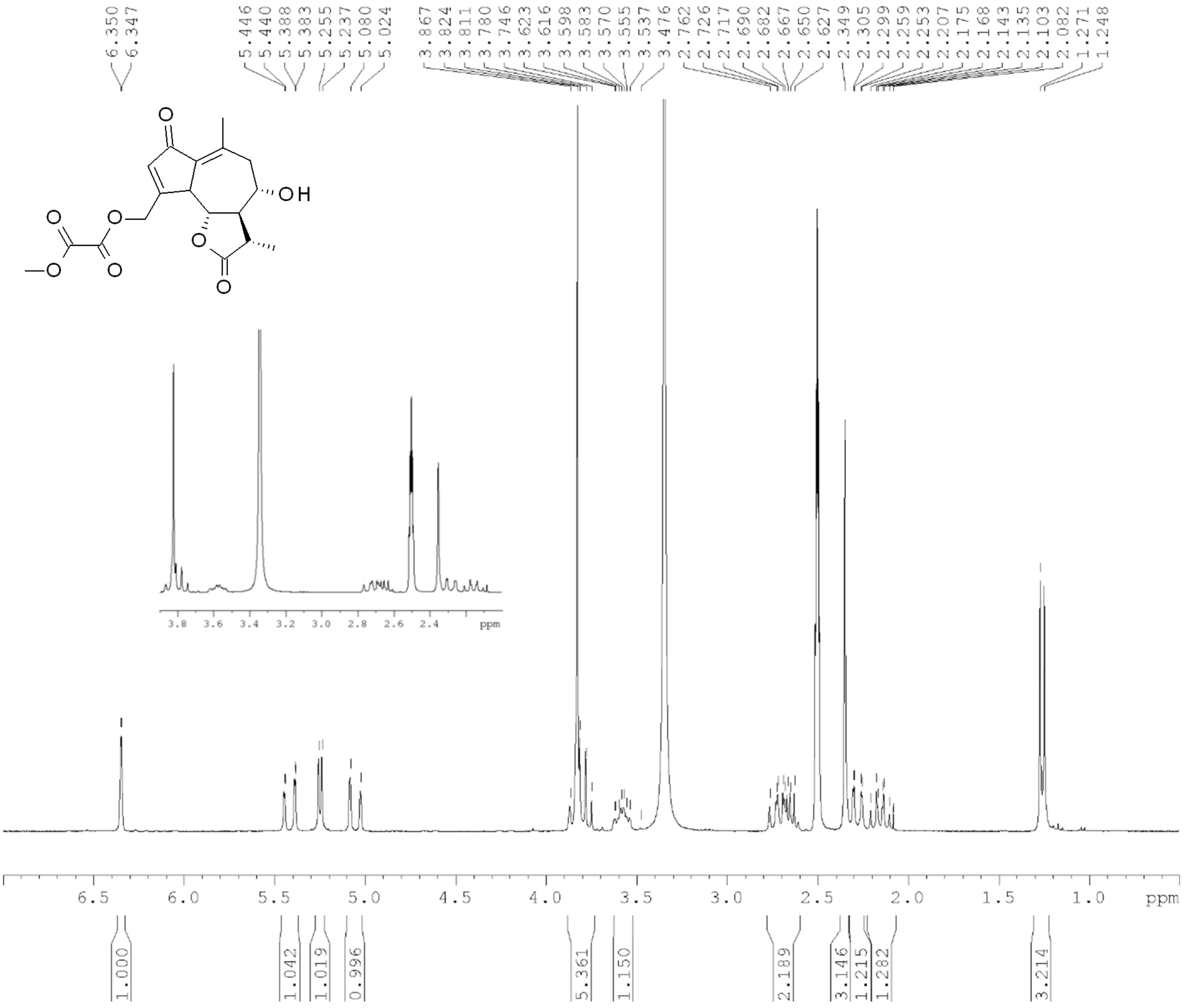

DHLc-Me-oxalate,^{13}C NMR (75 MHz, DMSO)

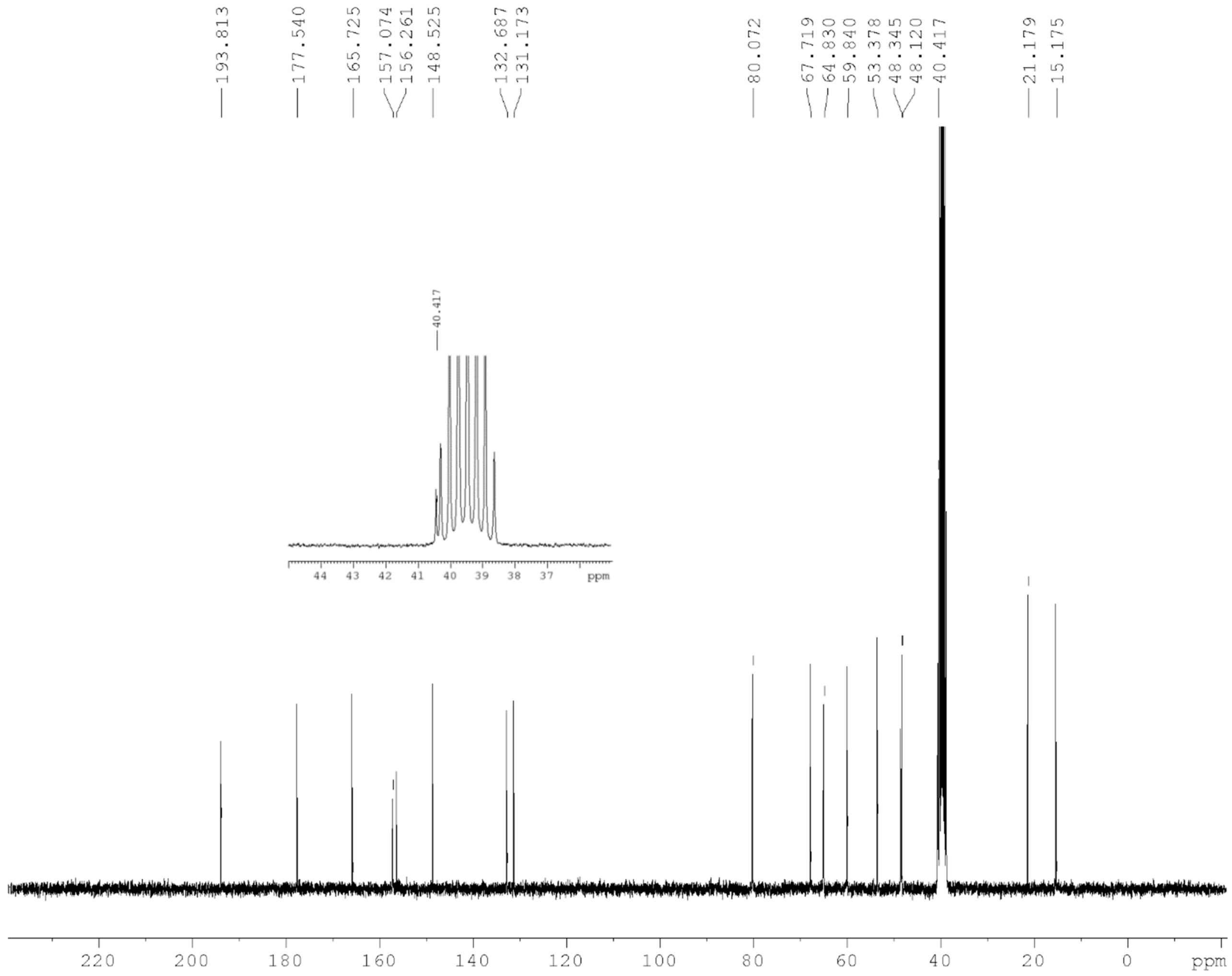

Lc-Me-oxalate, ^{1}H NMR (300 MHz, DMSO)

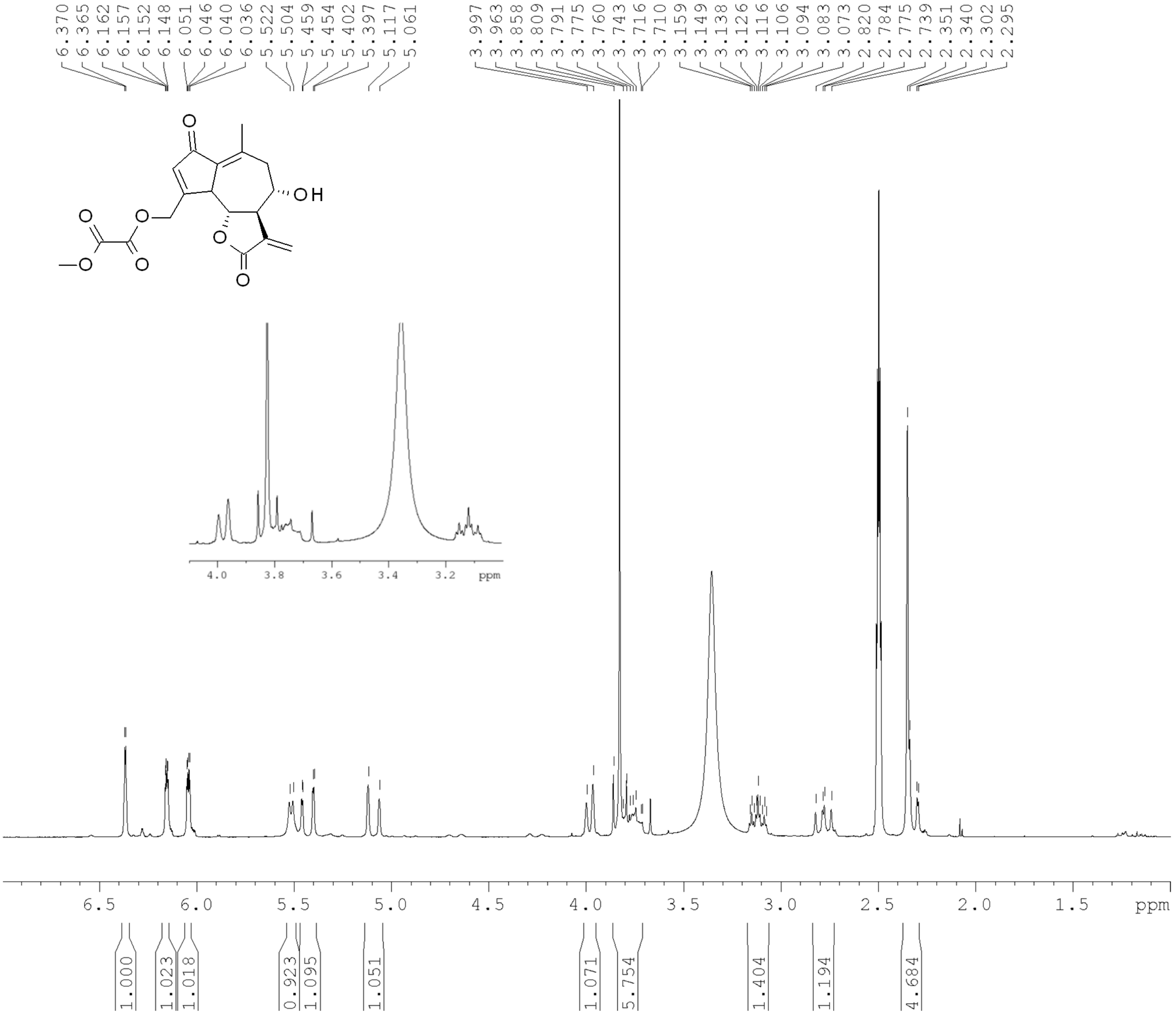

Lc-Me-oxalate,^{13}C NMR (75 MHz, DMSO)

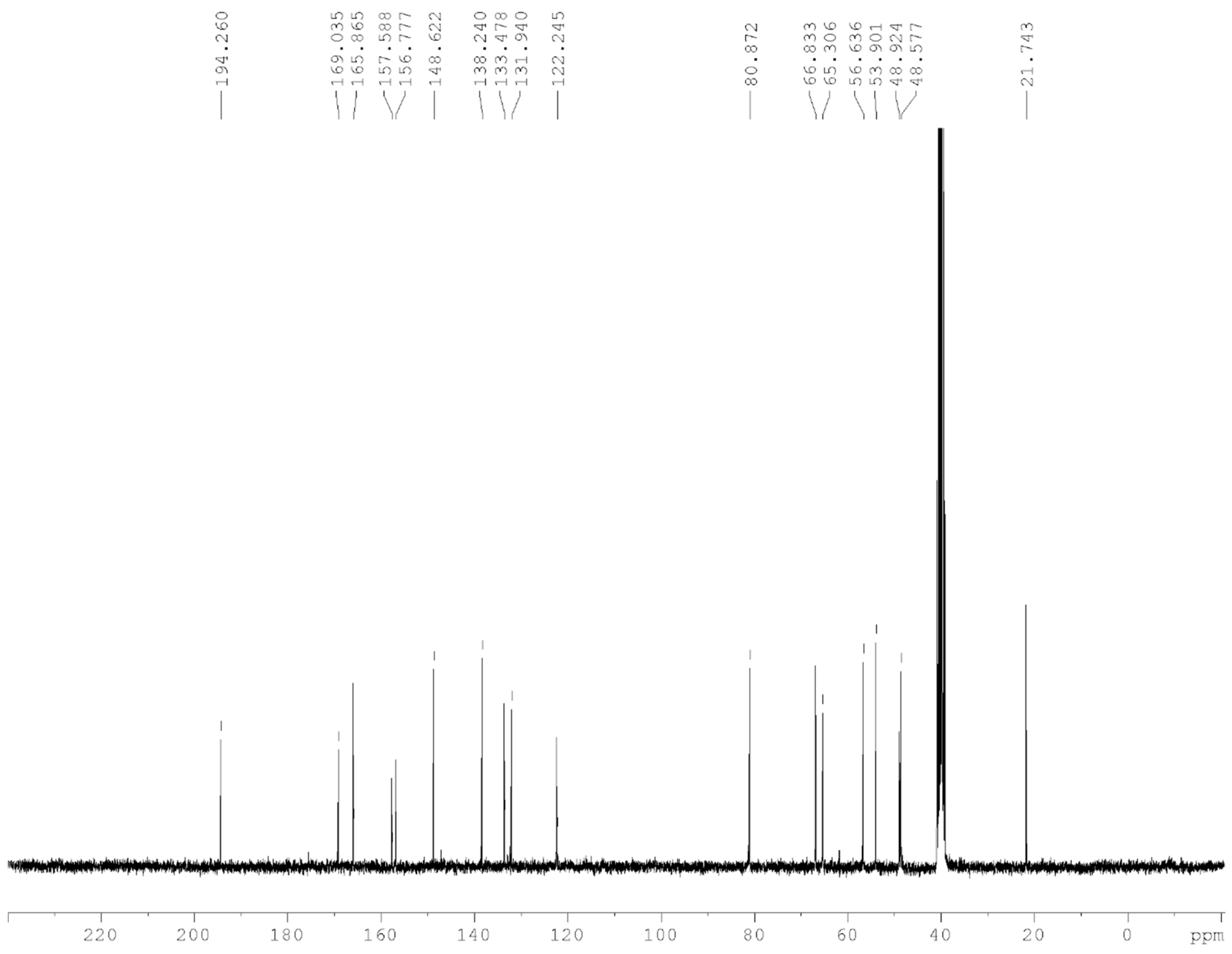

DHLc-oxalate, ^{1}H NMR (300 MHz, DMSO)

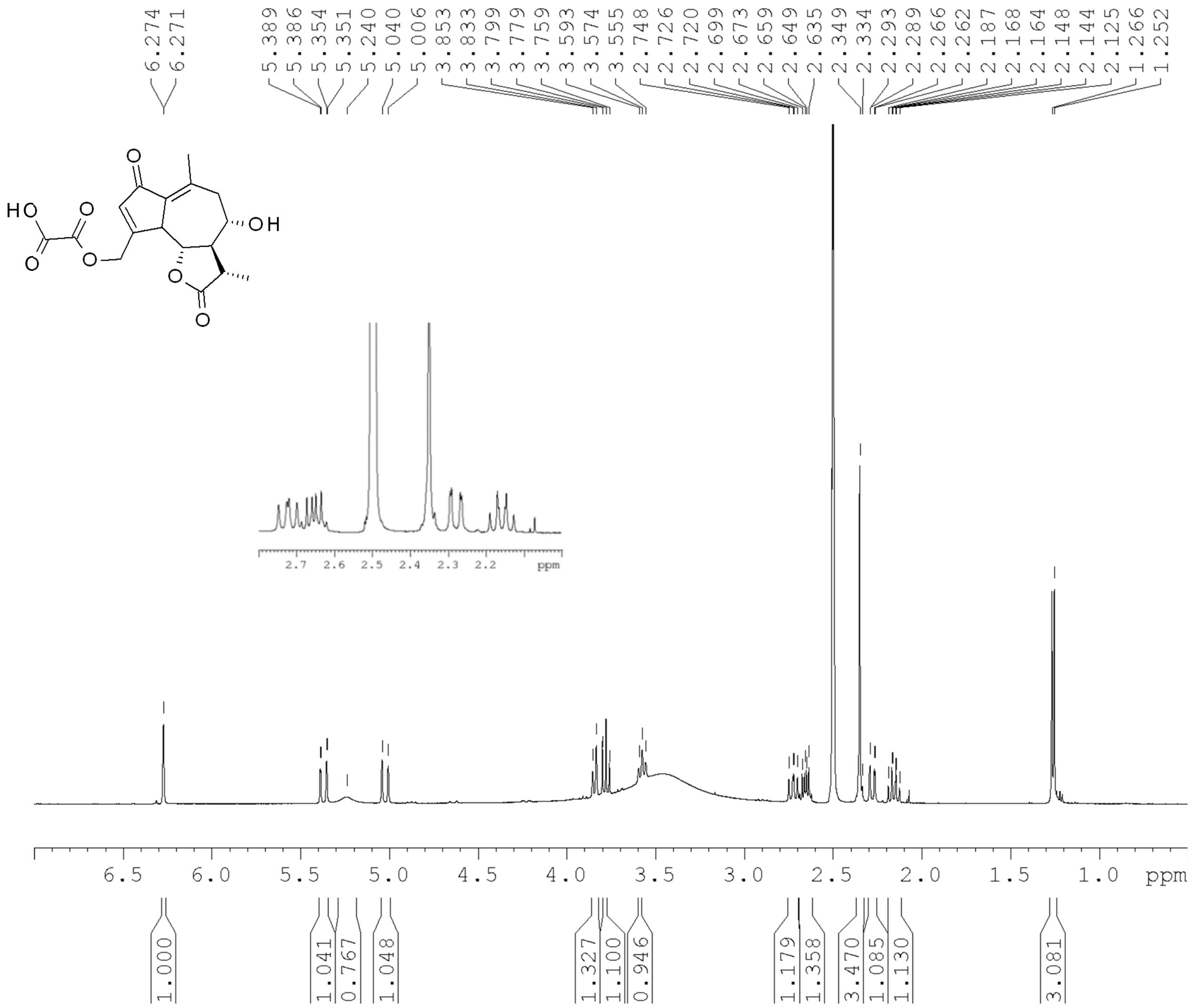

DHLc-oxalate,^{13}C NMR (75 MHz, DMSO)

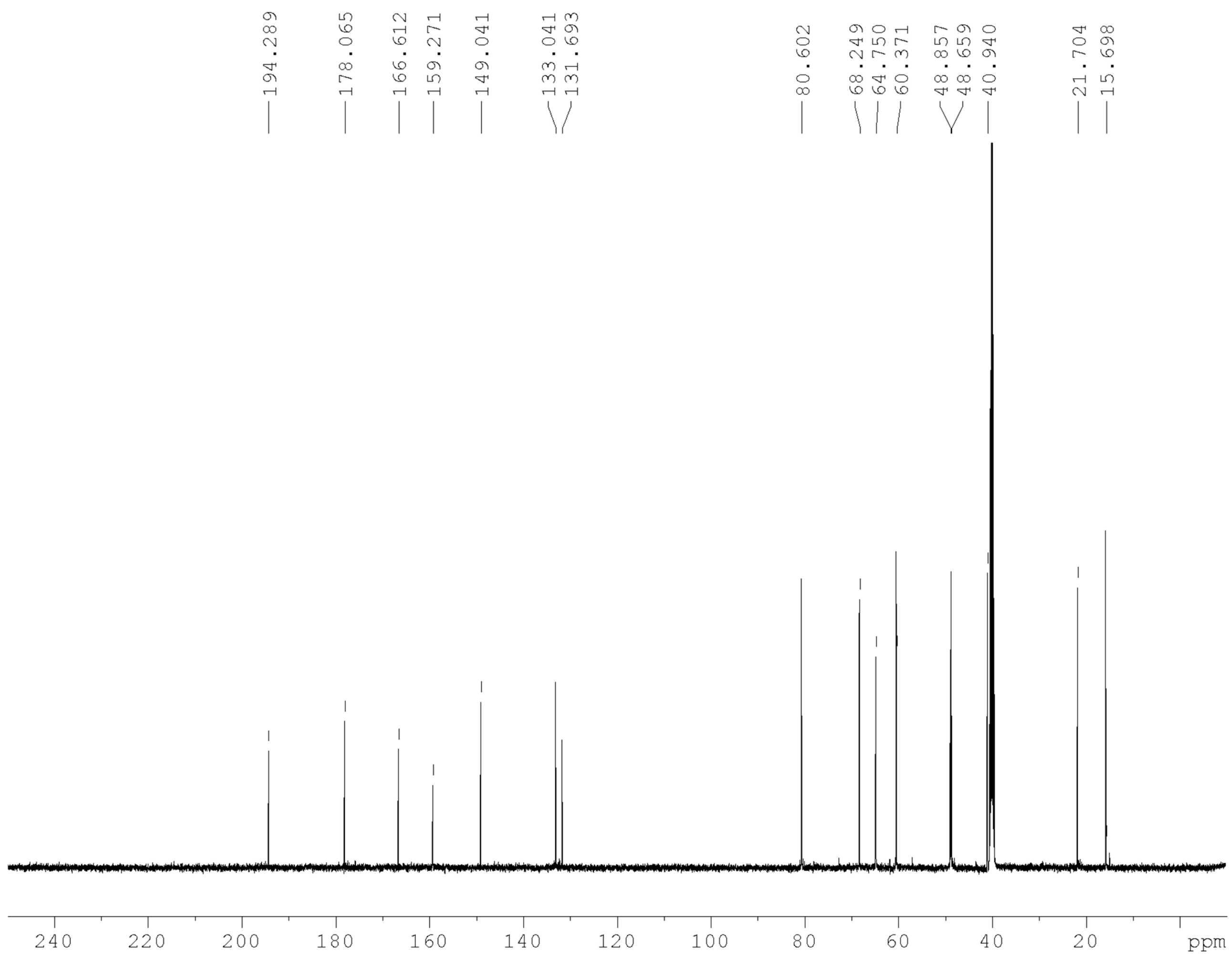

Lc-oxalate, ^{1}H NMR (300 MHz, DMSO)

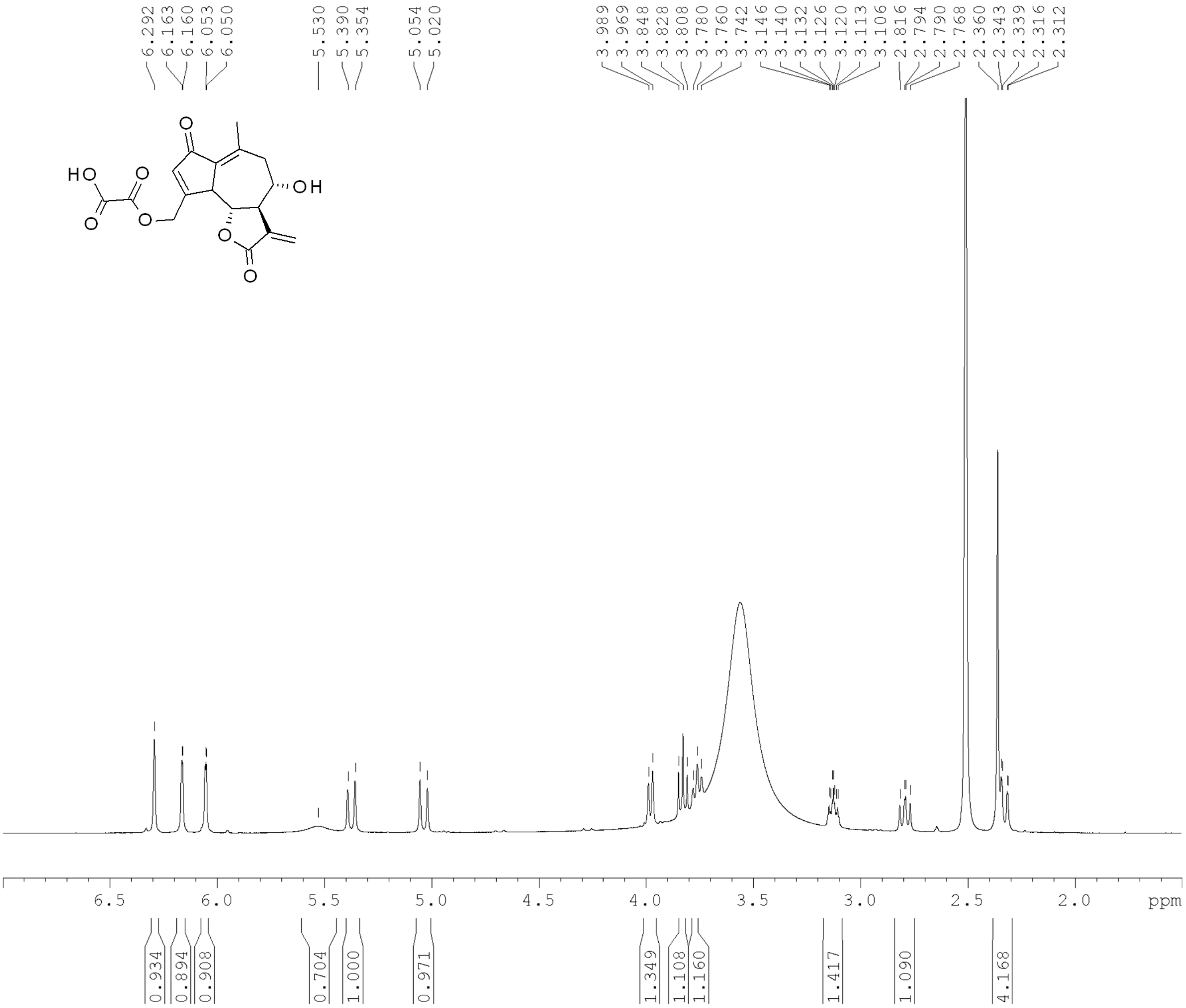

Lc-oxalate, ^{13}C NMR (75 MHz, DMSO)

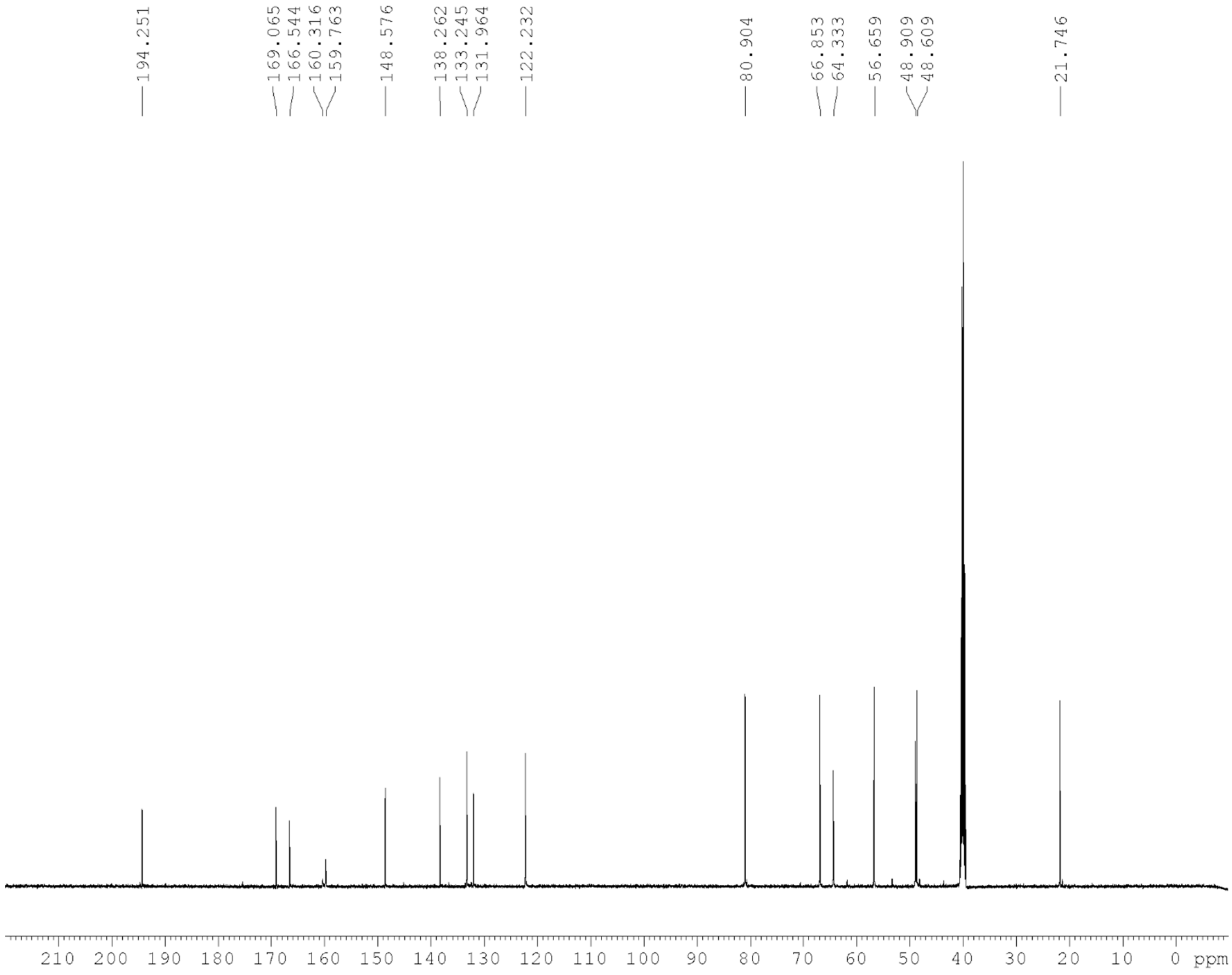

DHLc-glucoside, ^{1}H NMR (300 MHz, DMSO)

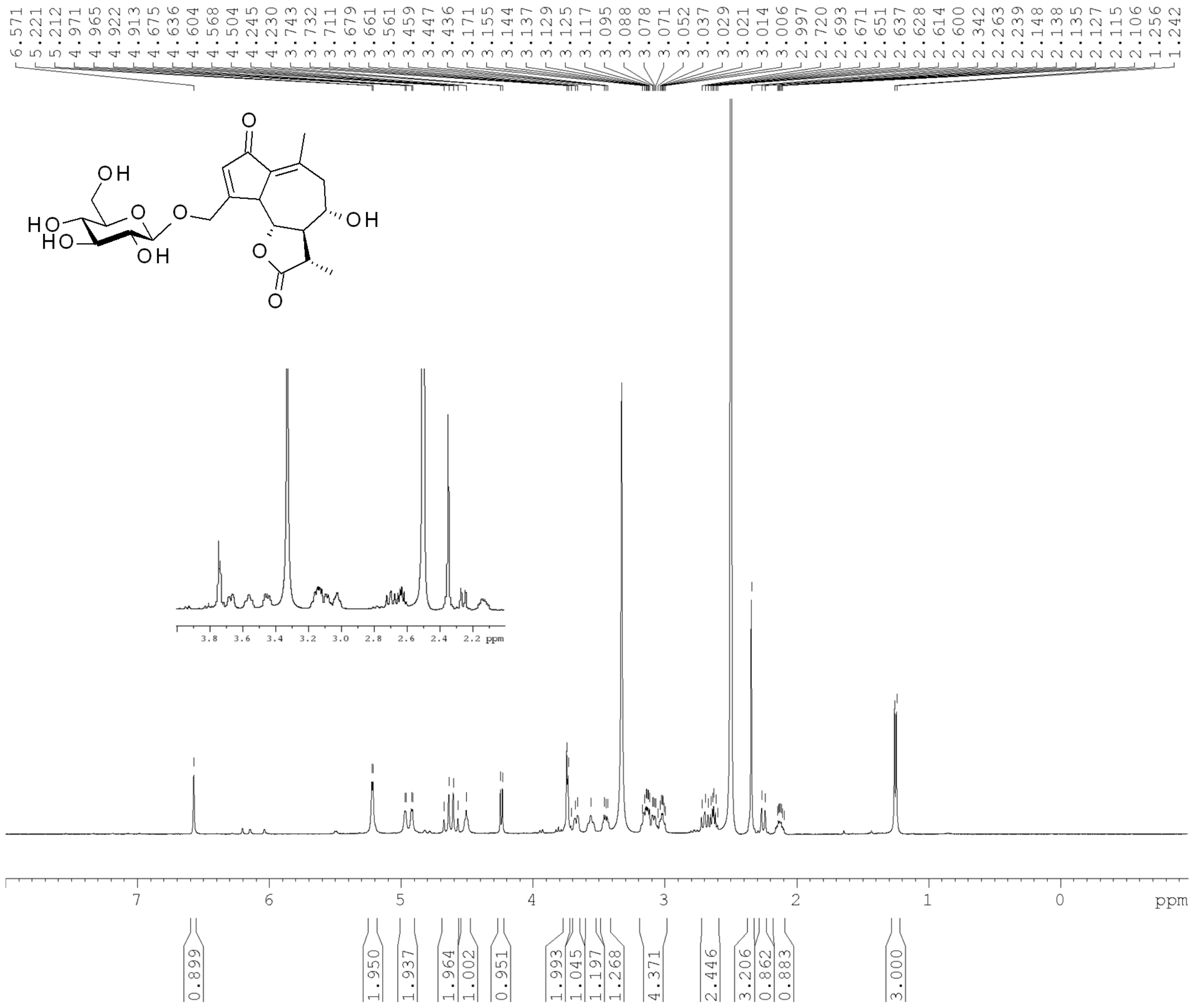

DHLc-glucoside, ^{13}C NMR (75 MHz, DMSO)

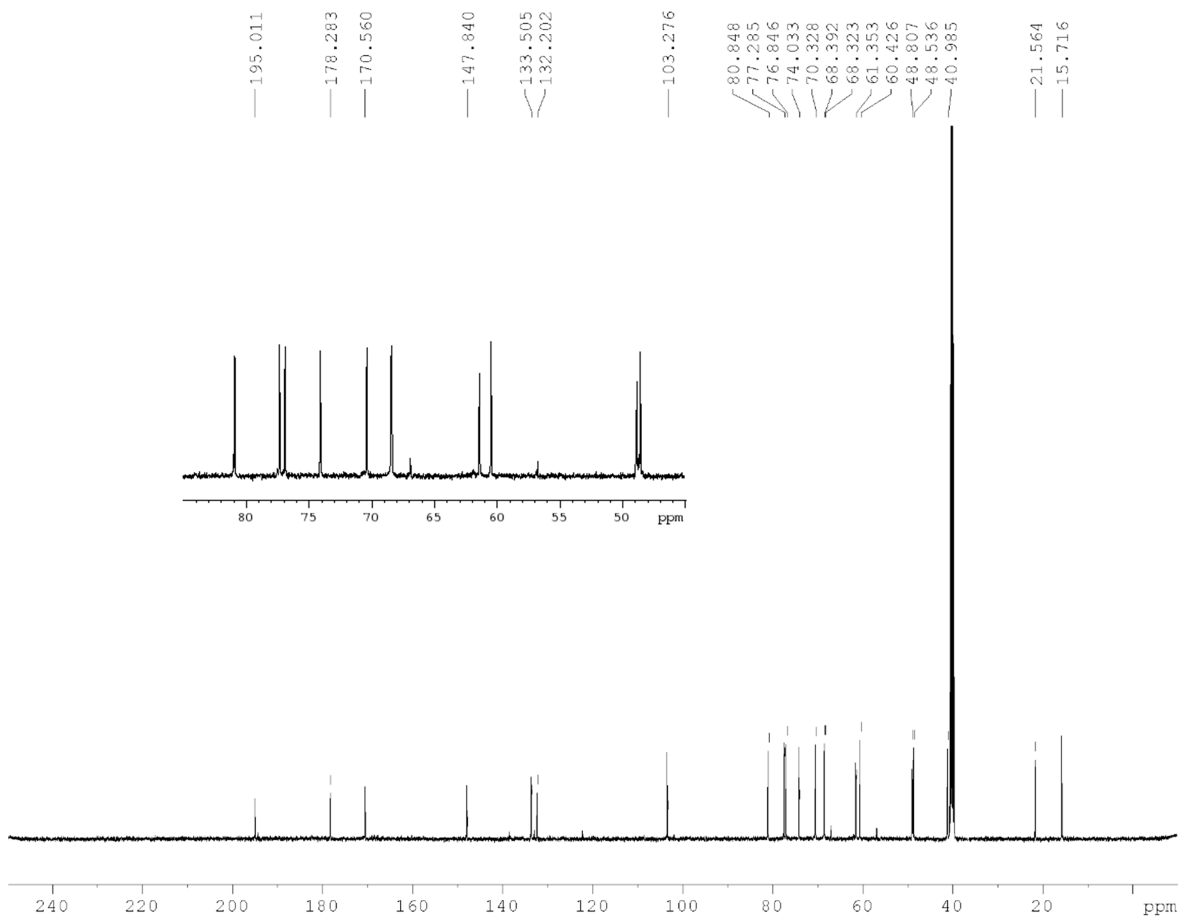

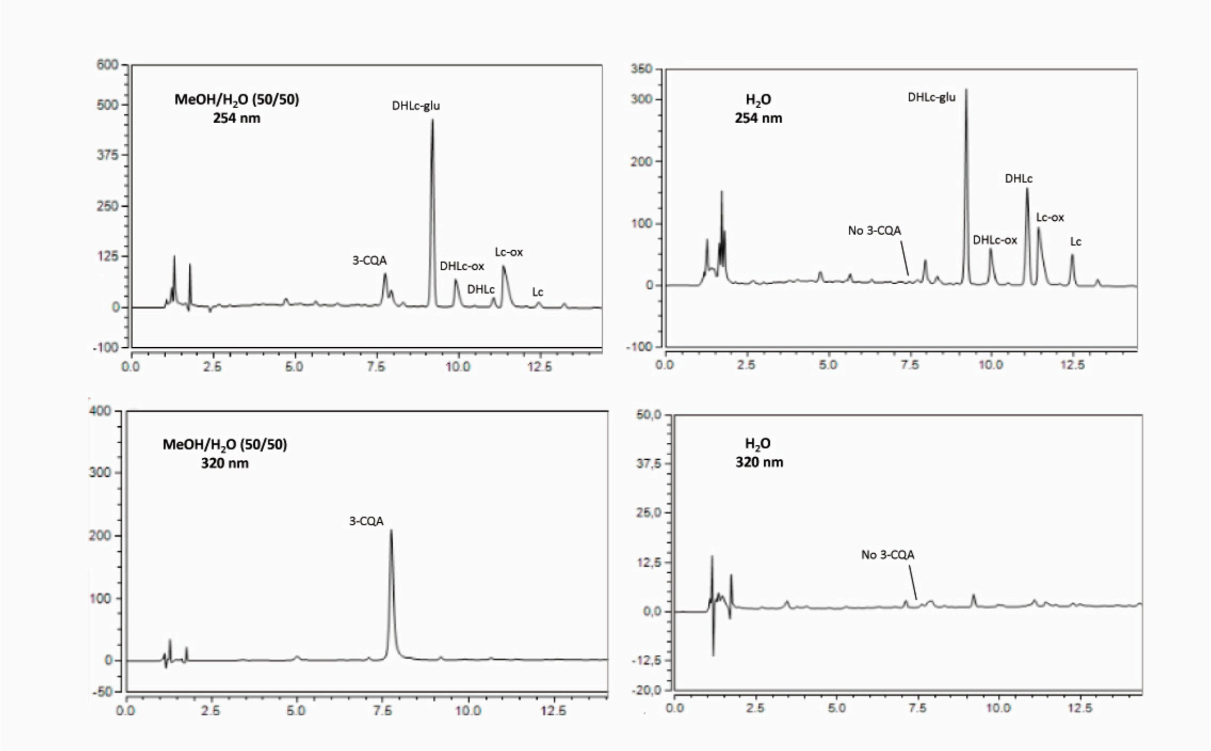

Figure S1. HPLC profiles (254 and 320 nm) of a water/methanol 50/50 and pure water extracts and different content in 3-CQA.

LC-QTOF-HRMS (+) Analysis of STLs

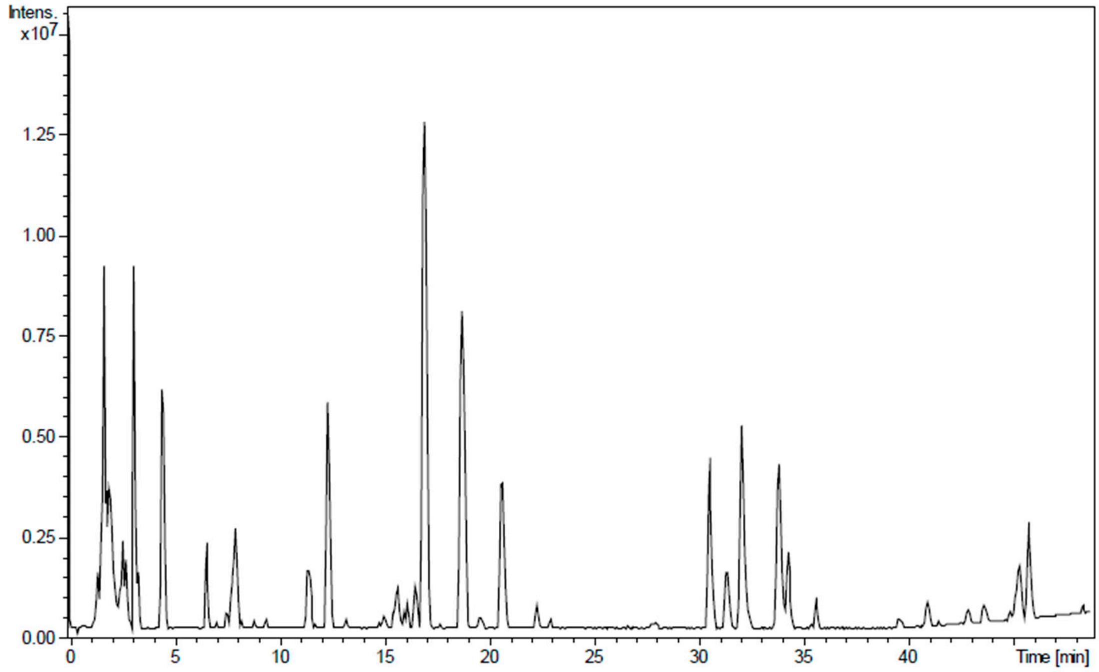

Figure S2. Base peak chromatogram (BPC, All − MS) of a water chicory extract obtained in the positive mode.

DHLc-glu

Na+ adduct 463 m/z
BPC

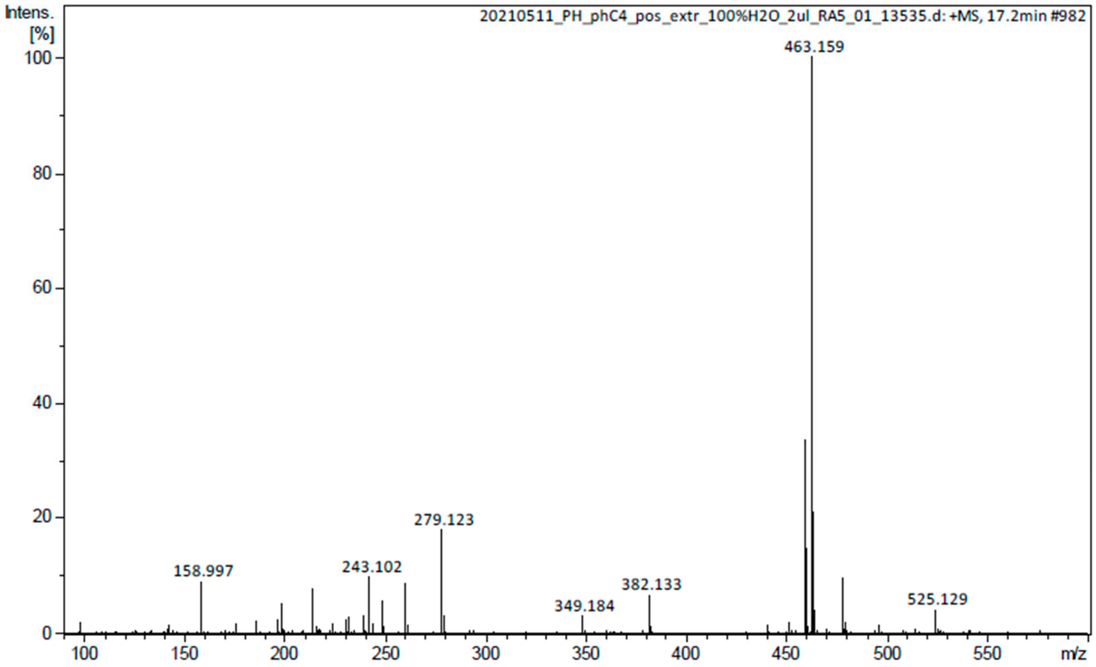

Na+ adduct 463 m/z
Zoom in

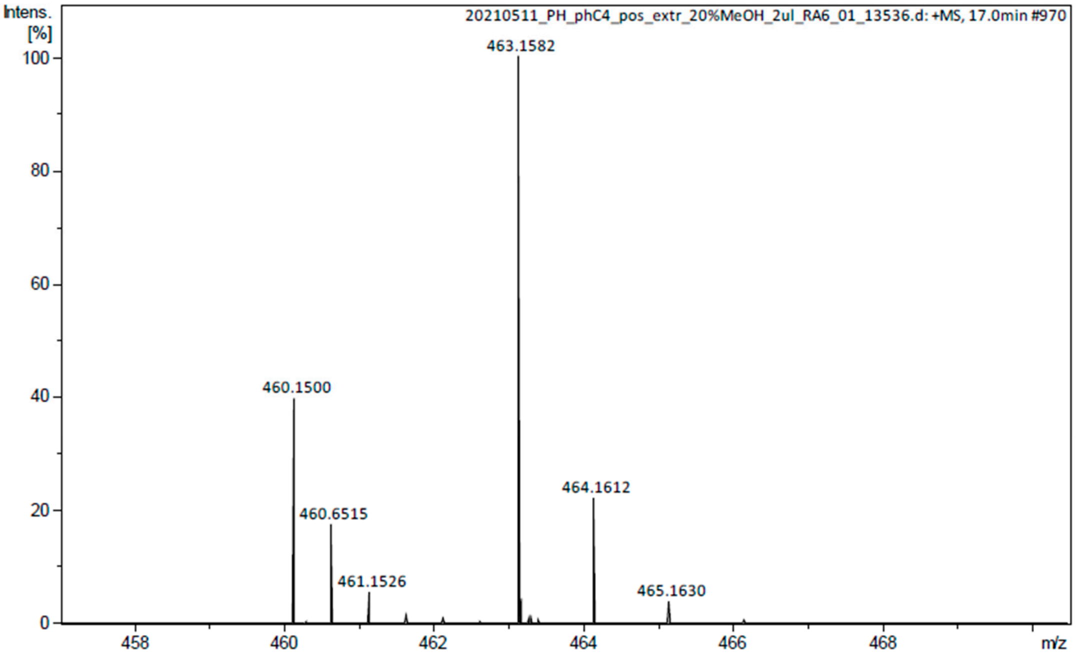

Zoom in : Na+ adduct 463 m/z; H+ 441 m/z

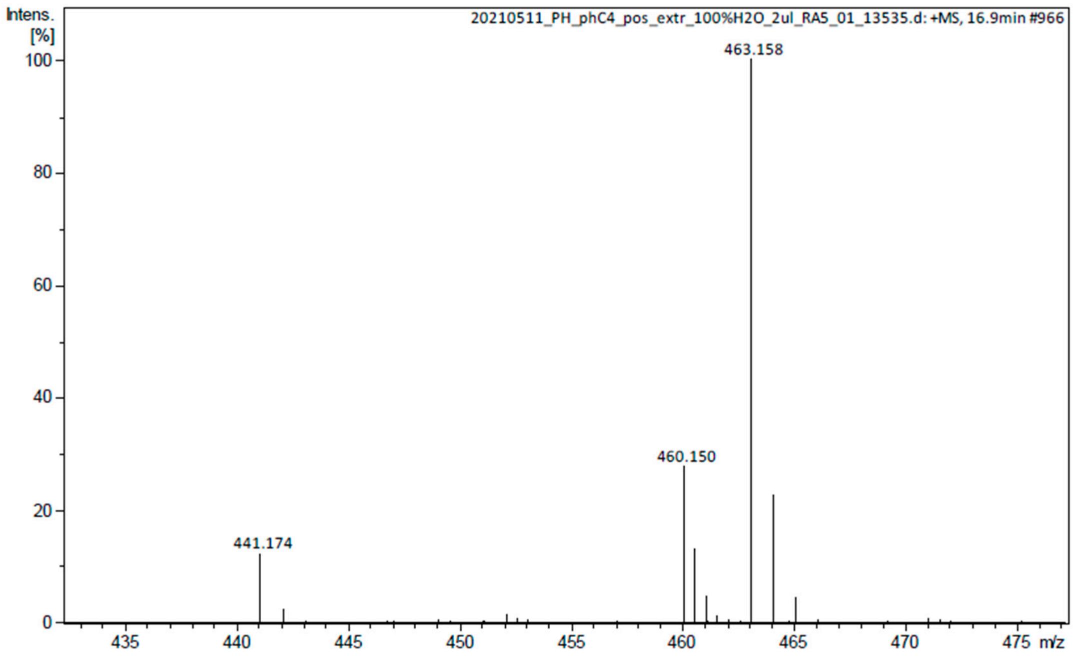

DHLc-ox

H+ 351 m/z

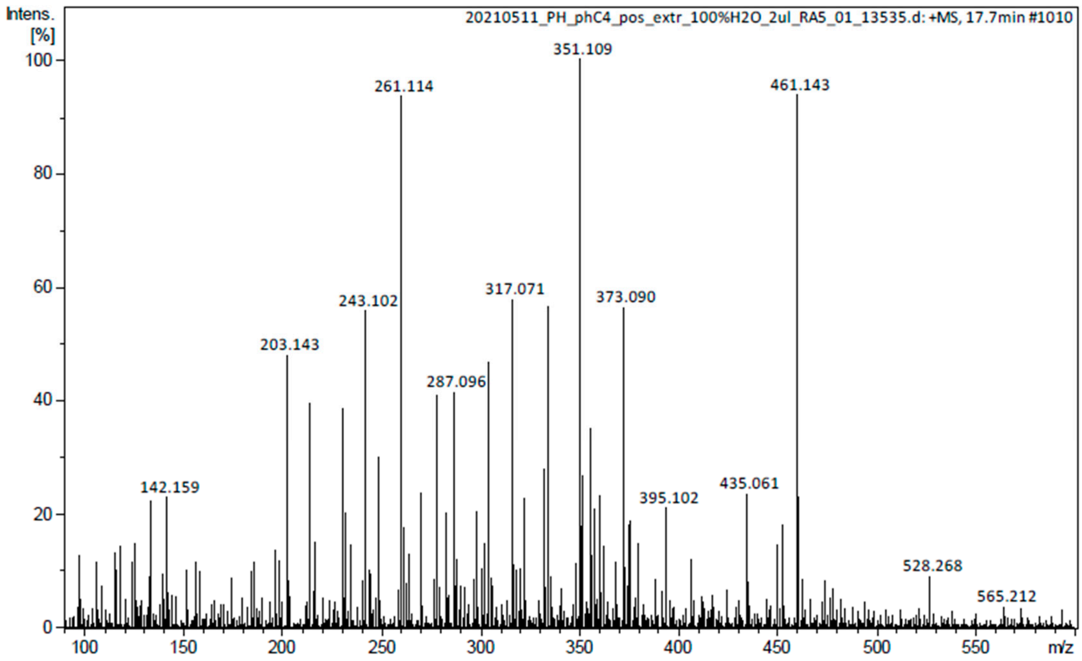

Zoom in : H+ 351 m/z

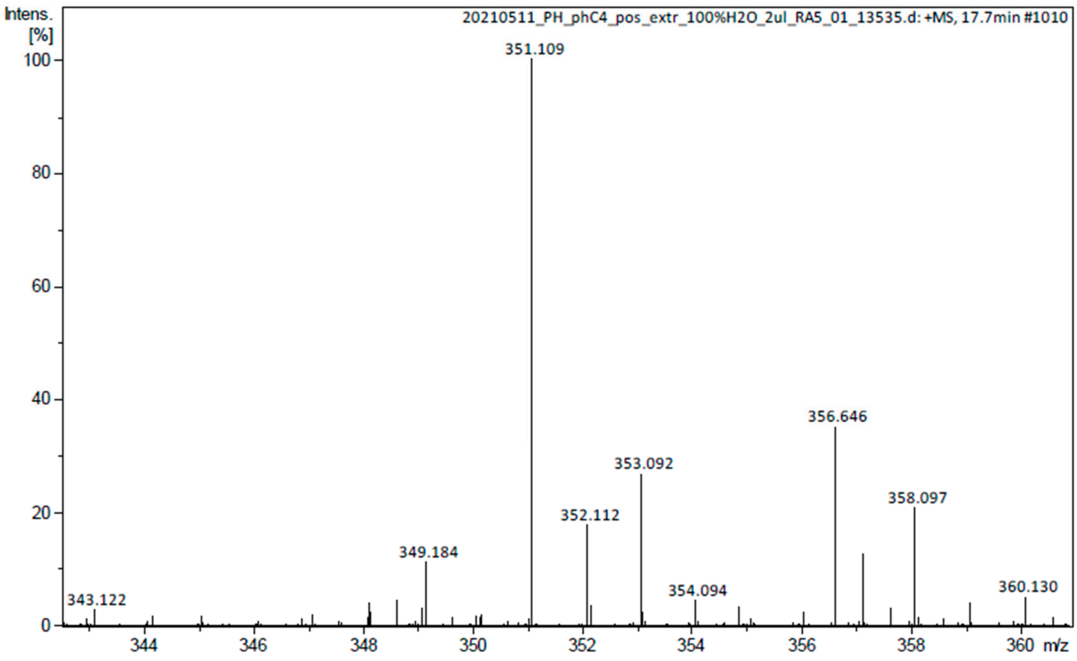

DHLc

Na+ adduct 301 m/z; H+ 279 m/z

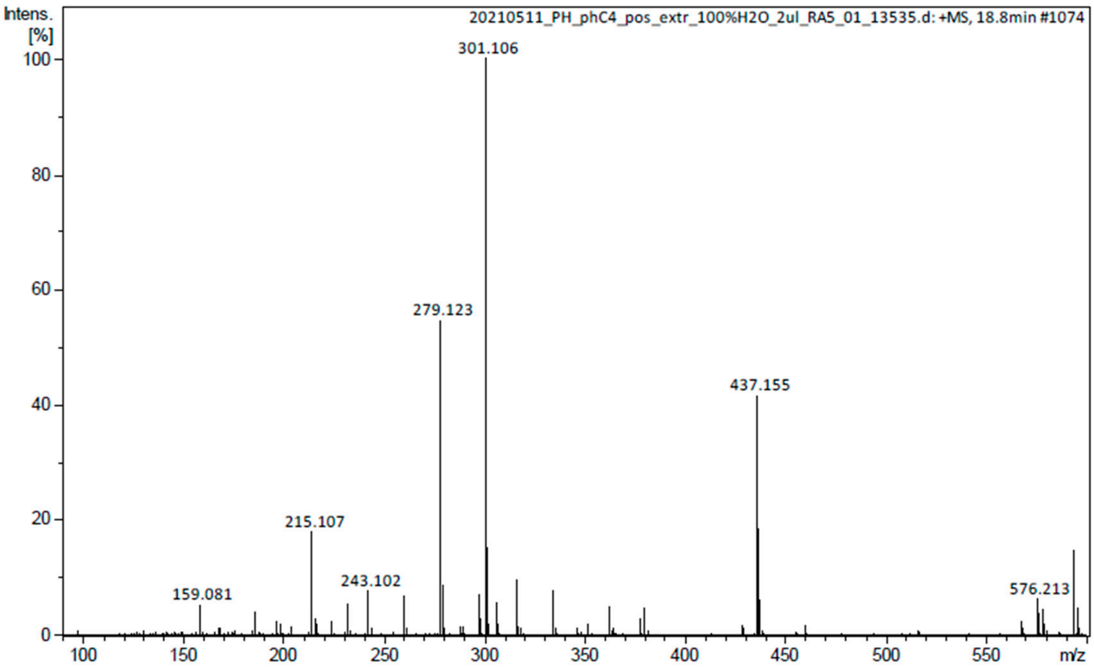

Zoom in : Na+ adduct 301 m/z; H+ 279 m/z

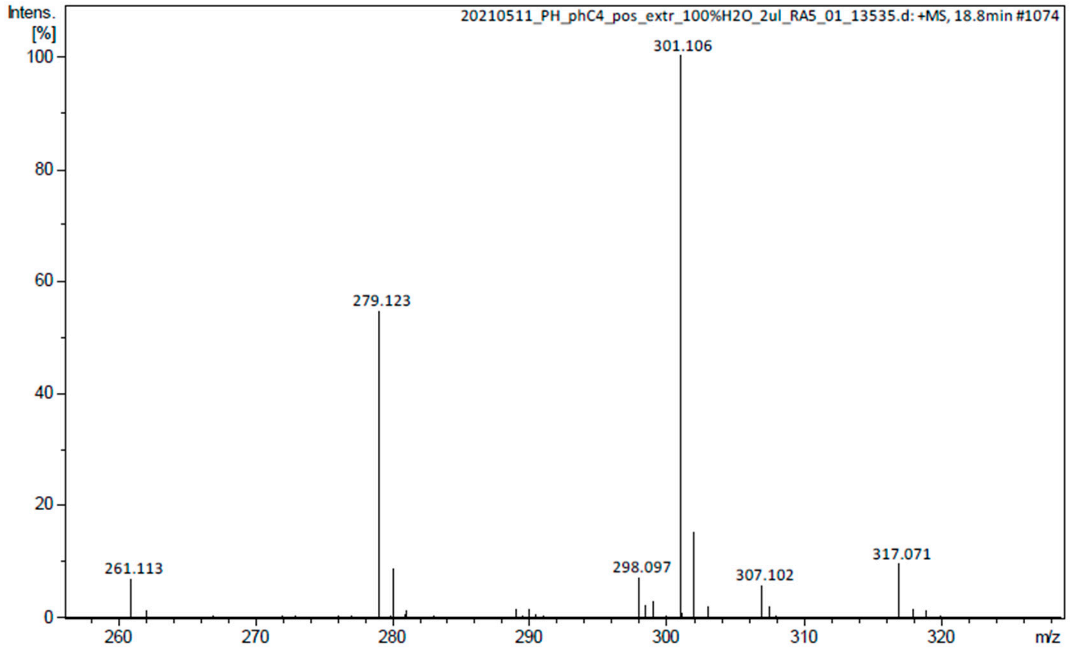

Lc-ox

Na+ adduct 371 m/z; H+ 349 m/z

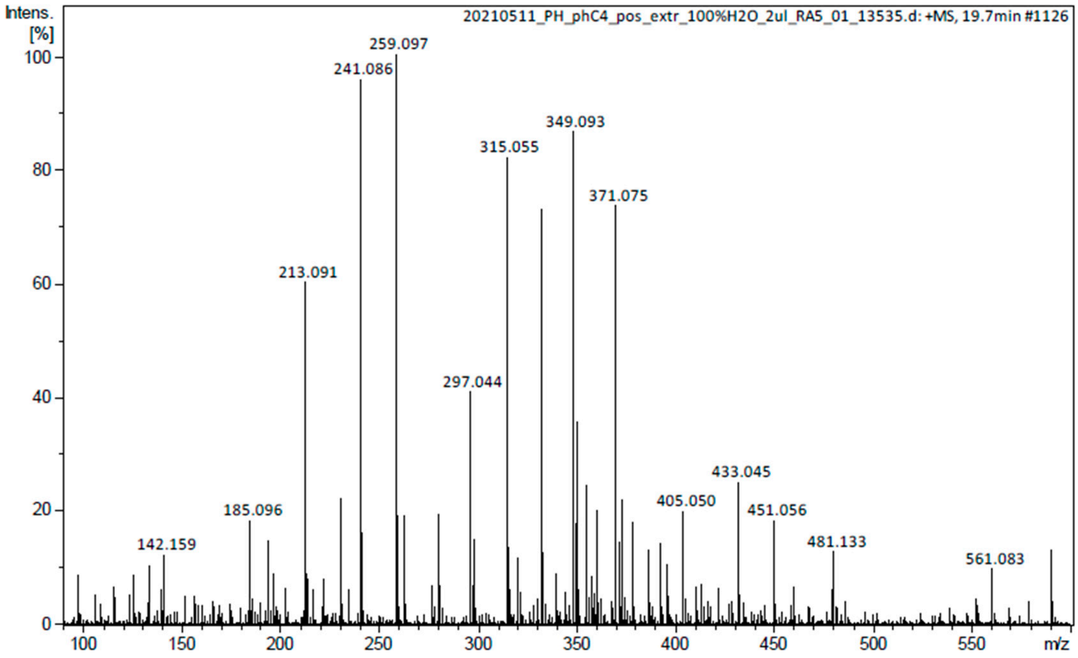

Zoom in : Na+ adduct 371 m/z; H+ 349 m/z

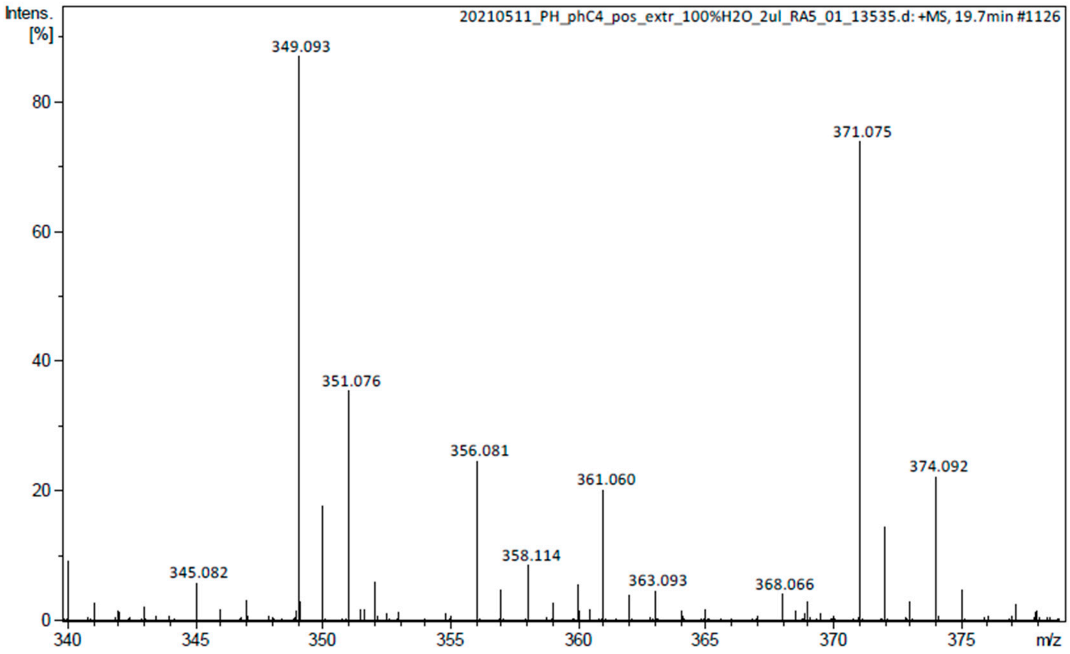

Lc

Na+ adduct 299 m/z; H+ 277 m/z

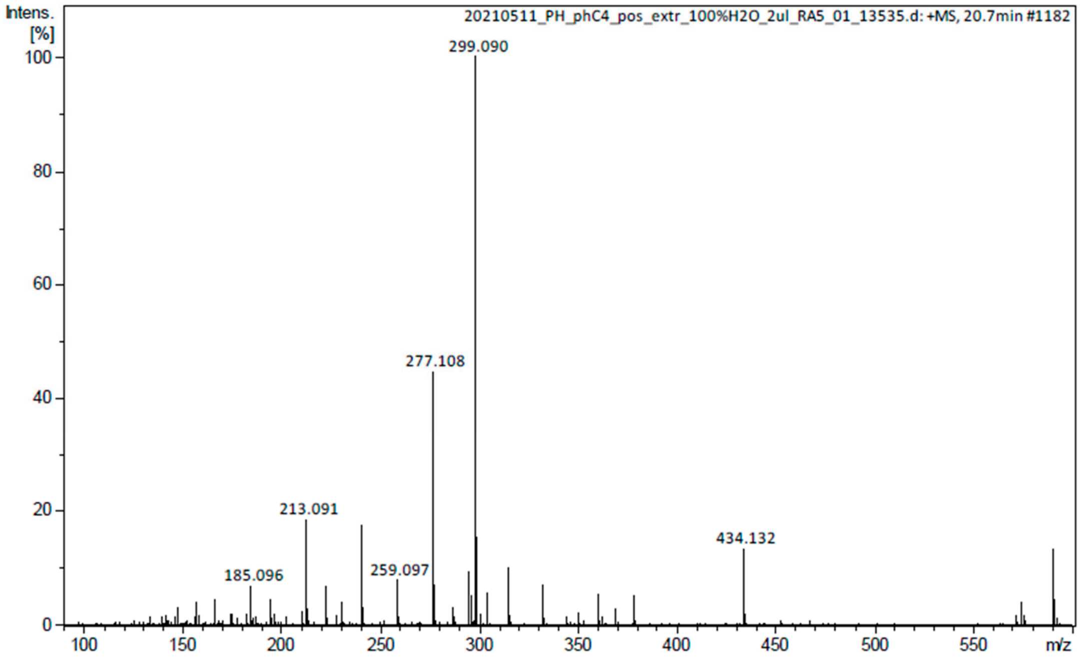

Zoom in : Na+ adduct 299 m/z; H+ 277 m/z

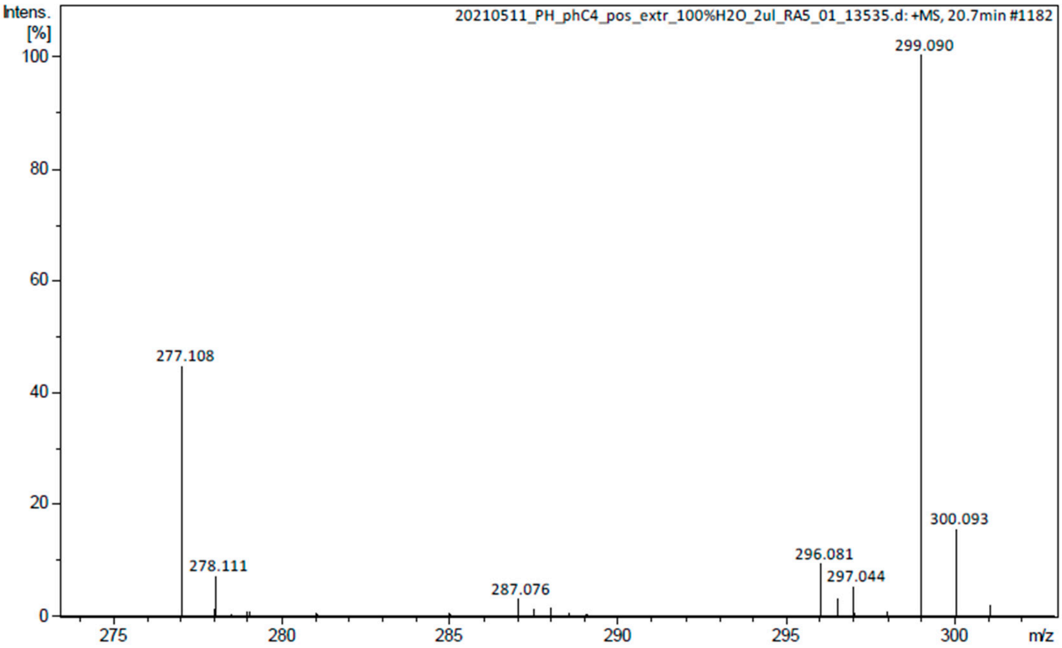

Supplement: Supplementary file 1 [file pharmaceuticals-16-00771-s001.zip › pharmaceuticals-2361427-supplementary.pdf]
